# Onset of circadian rhythmicity in the brain of Atlantic salmon is linked to exogenous feeding

**DOI:** 10.1371/journal.pone.0312911

**Published:** 2024-11-15

**Authors:** Mariann Eilertsen, Sissel Norland, David W. P. Dolan, Rita Karlsen, Ana S. Gomes, Charlotte M. Bolton, Herve Migaud, Ivar Rønnestad, Jon Vidar Helvik

**Affiliations:** 1 Department of Biological Sciences, University of Bergen, Bergen, Norway; 2 Department of Informatics, University of Bergen, Bergen, Norway; 3 Institute of Aquaculture, University of Stirling, Stirling, Scotland, United Kingdom; Karlsruhe Institute of Technology: Karlsruher Institut fur Technologie, GERMANY

## Abstract

An organism’s biological processes are adapted to and driven by rhythmicity in the natural environment and periodicity of light is one of the most influential factors. In a developing organism, the onset of circadian rhythmicity might indicate the time point of functional necessity for aligning processes to the environment. Here, the circadian clock mechanism has been studied in the developing brain of Atlantic salmon (*Salmo salar*), by comparing the endogenous feeding alevin, independent on the environment for nutritional supply, to the exogenous feeding fry, dependent on the light period for detecting and catching prey. The results showed that while only a few clock genes were cyclic in the yolk sac alevins, many of the clock genes and genes of the circadian rhythm pathway cycled significantly in the feeding fry. Few genes were differentially expressed between time points in the circadian sampling series during the yolk sac stage, but several hundred genes were found differentially expressed in the first feeding stage. Genes important for cell cycle progression were cyclic or differentially expressed between time points after exogenous feeding, indicating a clock-controlled cell cycle at this stage. The expression of important genes in the melatonin synthesis were also cyclic in the feeding fry with an acrophase in the transition between light and dark or in darkness. Analyzing the impact of exogenous feeding on the developing brain supported a shift from utilization of proteins and lipids in the yolk to utilization and allocation of dietary energy and nutrients. Taken together, the life history transition related to onset of exogenous feeding is linked to the establishment of a persistent circadian rhythmicity in the salmon brain, which needs to be synchronized to light-dark cycles to enable the fry to search and capture feed.

## Introduction

In most organisms, biological time keeping is essential for synchronizing behavior, physiology, and gene expression to circadian and circannual rhythms. Daily solar cycles of light and dark are the most predictable environmental cues to modulate these rhythms, perceived by non-image forming photoreceptors [1, 2]. The circadian rhythm is driven by oscillations in gene expressions with a periodicity of about 24 hours, which is a result of molecular interactions of “clock genes”. There has been a great effort to understand the mechanisms of the circadian clock, mainly using fruit fly (*Drosophila melanogaster*), mouse (*Mus musculus*) and zebrafish (*Danio rerio*) as model organisms [3, 4].

The mammalian molecular core clock mechanism is based on positive and negative transcriptional-/translational-feedback loops where the positive elements circadian locomotor output cycles kaput (*Clock*) and aryl hydrocarbon receptor nuclear translocator-like (*Arntl*) (also referred to as brain and muscle arnt-like (*Bmal*)) drive the expression of the negative elements, period (*Per*) and cryptochrome (*Cry*), which in turn inhibit the transcriptional activation driven by the positive elements [4–6]. The output of the circadian clock is the rhythmic regulation of clock-controlled genes that are either directly or indirectly controlled by the heterodimer formed by the CLOCK and ARNTL proteins, leading to circadian downstream pathways and events [5, 7]. The clock mechanism also consists of a stabilizing loop of genes (RAR-Related Orphan Receptor (*Ror*), nuclear receptor subfamily 1 group d (*Nr1d*, also referred to as *Rev*-*Erb*) and casein kinase epsilon/delta (*Csnk1ε/δ*)) which interact with the core clock to either promote or repress heterodimer interactions [8]. Transcriptional regulation of clock genes and clock-controlled genes is the result of three interlocked transcriptional feedback loops acting on *cis*-elements (E-boxes, ROREs and D-boxes) in the promoters and enhancers of the target genes. The direct regulation of transcription is through binding of the CLOCK:ARNTL heterodimer to the E-boxes while the indirect regulation is a result of proteins binding to the ROREs and D-boxes, respectively [8]. In mammals, the light input pathway resetting the clock mechanism is through a light-induced expression of *Per1* and *Per2* [9, 10], while in zebrafish *per2* and *cry1a* have been shown to play a key role in light entrainment [11, 12]. Light-evoked expression of *per2* in zebrafish involves E- and D-box elements close to the transcriptional start site, where the E-box controls circadian clock regulation by Clock:Arntl activity and the D-box promotes light-driven expression through binding of the light-inducible transcription factor thyrotroph embryonic factor (Tef) [13]. Promoter analyzes of *cry1a* have shown that a single D-box serves as the key light-responsive element suggesting that the D-box is a general convergence point for light-driven signaling [14]. Further, the period length of oscillation depends on the stability of the Per and Cry repressors and is regulated by their phosphorylation states and E3 ubiquitin ligase pathways [8].

Melatonin is among the major outputs of the vertebrate circadian clock mechanism. Rhythmic information is given to the organism through daily variations of secretion of melatonin into the blood and cerebrospinal fluid from the pineal gland/organ, with high concentration at night and low during the day [15]. In contrast to mammals, the pineal organ in teleosts is directly light sensitive and has dual functions as photoreceptor and endocrine tissue [15, 16]. In most teleosts, melatonin secretion involves the circadian clock mechanism, where the nocturnal rise in melatonin is a result of an increased expression of the clock-controlled gene arylalkylamine N-acetyltransferase (*aanat*), which is the first enzyme in a two-step process transforming serotonin to melatonin [17]. In salmonids, it is suggested that the circadian regulation of melatonin production was lost and that it is only controlled by light signals in an on/off manner, since the melatonin release did not exhibit circadian rhythms after a switch from light-dark (LD) cycles to constant darkness [15, 18]. In addition, circadian studies of *aanat* expression in the pineal of rainbow trout (*Oncorhynchus mykiss*) and Atlantic salmon showed a non-rhythmic expression pattern in contrast to other teleosts [19, 20] and *in silico* analyzes of the promoter region in salmon indicated lack of the E-box elements, suggesting a loss of functional connection between the circadian system and melatonin production [20].

In teleosts, the onset of circadian rhythmicity has been studied in a few species such as zebrafish, medaka (*Oryzias latipes*), Senegalese sole (*Solea senegalensis*) and Mexican blind cavefish (*Astyanax mexicanus*), revealing that cycles of light and dark are required for rhythmic expression of clock genes in the developing embryos and larvae [21–24]. In zebrafish *per* and *cry* oscillate within 24 hours post fertilization (hpf), while *clock* and *arntl* first start to oscillate at 96 hpf [22]. The rhythmic transcription of *clock* and *arntl* correspond to the establishment of circadian clock output processes, such as light-entrained rhythmic locomotor activity and timing of DNA replication [25, 26]. In medaka, *per* and *cry* genes also start to oscillate early, while *clock* and *arntl* begin to cycle at the time of hatching (12–14 days post fertilization (dpf)) [21]. Further, acceleration in development by increased temperature or removal of chorion have been shown to advance the onset of rhythmicity in *clock* and *arntl* expression, indicating that the maturation of the circadian clock is dependent on the developmental stage and time of hatching [21]. In salmonids, which have a long developmental window and hatched alevins with a large yolk sac of endogenous nutrients, the onset of circadian rhythmicity has only been studied for a few clock genes, showing oscillation of *per1* at 8–9 dpf and *clock* at 42–43 dpf in rainbow trout [27]. However, high quality genomic and transcriptomic data have advanced studies on clock genes, clock-controlled genes, and circadian clock output processes. Microarray analyses of feeding larvae of gilthead sea bream (*Sparus aurata*) have shown differential expression of core clock genes and a diurnal activation of pathways related to phototransduction, intermediary metabolism, development, chromatin remodeling and cell cycle regulation [28]. Zebrafish larvae raised in darkness and then exposed to a light pulse, had an enrichment of genes involved in circadian rhythms, stress response and DNA repair [29] and a 48 h circadian sampling series in whole zebrafish larvae showed that most clock genes were cyclic before first feeding, oscillating with an anti-phase relationship between zebrafish and mice [30]. In contrast, a recent study of whole Atlantic salmon alevins showed that many clock genes were expressed early in development but that only two genes were cyclic before first feeding [31].

The life history transition of salmon is closely linked to changes in the seasonal cycling of light period and processes such as hatching and emerging of alevins at first feeding [32], smoltification [33] and reproduction [34] are synchronized to specific periods of the year. In nature, spawning takes place in the rivers in late autumn or early winter and the embryos develop over the winter and hatch in the spring [32, 34]. The alevins live in the gravel and utilize endogenous yolk nutrients until the yolk sac is nearly depleted when they emerge from the protective gravel and start exogenous feeding in the river [32]. This life history transition is intriguing in relation to circadian rhythmicity since the organism shifts from a life with a constant supply of yolk nutrition to a lifestyle of active search and catch of prey under daylight conditions as a visual feeder. In this study, RNA sequencing of circadian sampling series of Atlantic salmon brains at two developmental stages, before (720 dd (day degrees)) and after the onset of exogenous feeding (920 dd), have been done, aiming to reveal a potential onset of circadian rhythmicity around this important life history transition. By taking a global approach, clock genes and key genes in circadian rhythmicity have been studied, together with clock-controlled genes and circadian clock output processes such as the cell cycle and melatonin production. The results, following the gene expression over 48 h, verified that only a few clock genes cycled in the brain during endogenous feeding, but the brain of the visual feeding fry, had an advanced circadian clock mechanism, with 26 cycling clock genes and a rhythmic activation of clock-controlled genes.

## Materials and methods

### Ethical statement

The study was done in a facility given approval by the Norwegian Food Safety Authority (VSID2135) and complied with the ARRIVE guidelines [35]. As the alevins and fry did not undergo handling except for euthanasia, special approval was not required according to Norwegian National legislation via the Norwegian Animal Welfare Act (LOV-2009-06-19) and Regulations on the Use of Animals in Experiments (FOR-2015-06-18-761), given by EU (Directive 2010/63/EU) for animal experiments. All alevins and fry were euthanized with an overdose of metacaine (MS-222TM; MSD Animal Health, Netherlands) on-site, before further handling.

### Animals, experimental design and sampling

The study was performed on two sibling groups of Atlantic salmon (*Salmo salar*) (broodstock eggs and sperm from two females and two males) were obtained from the aquaculture industry, Mowi, Tveitevågen, Norway. The euthanasia and subsequent sacrifice of the fish were done by the aquaculture farmer on field site according to the Regulation on slaughterhouses and production facilities for aquaculture animals (FOR-2014-12-15-1831). Unfertilized eggs and sperm were transported to the laboratory facility at the High Technology Center, University of Bergen, Norway, where the fertilization, rearing and circadian sampling series also took place. The fertilized eggs were incubated in a LD cycle of 14:10 from fertilization to the end of the experiment. The light was switched on and off at 8 am and 10 pm, respectively, with an incremental transition period of 30 min when changing between light periods. The eggs were incubated in two individual egg incubator chambers (each incubator contained 3 individual racks). The light was applied by a state-of-art light-emitting diode (LED) technology (Signify, The Netherlands) and the light intensity was 0.1 W/m^2^ (measured 2 cm below water surface) with a wavelength range from 414–781 nm and a ~ λmax at 610 nm (see [31] for more details on the light measurements and the spectral properties). The incubation temperature was 5.7 ± 0.5°C. The developmental stage of the alevins and fry was calculated by day degrees (dd) (day x degree). For an accurate calculation, the mean temperature each day was determined by temperature measurements every 10^th^ minute, and then added up day by day from fertilization to the sampling point. Before first feeding, at approx. 712–723 dd (here named 720 dd), a 48-hour circadian sampling series was carried out at 4 h intervals (12:00 time point (TP) 1, 16:00 (TP1), 20:00 (TP1), 00:00 (TP1), 04:00 (TP1), 08:00 (TP1), 12:00 (TP2), 16:00 (TP2), 20:00 (TP2), 00:00 (TP2), 04:00 (TP2), 08:00 (TP2), 12:00 (TP3) corresponding to zeitgeber time (ZT) ZT4 (TP1), ZT8, ZT12, ZT16, ZT20, ZT0, ZT4 (TP2) …. ZT4 (TP3). Four individual alevins were randomly sampled from the racks at each sampling point. The alevins were euthanized with an overdose of MS222, and the heads were cut off and frozen directly in liquid nitrogen. At 730 dd the alevins were transferred and randomly allocated to three feeding tanks. The light intensity was increased to 1.0 W/m^2^ (measured 2 cm below water surface) in the feeding tanks to compensate for the increased water level (45 cm) in the feeding tanks. The temperature was gradually increased to 11.5°C after transfer and before first feeding. The fry was fed a commercial diet (0.6 mm, EWOS Micro starter diet, Norway) twice a day (09:00 and 16:00) from 772 dd. The daily feed given was 4% of the body weight (in addition to 25% extra) and 25% of the food was given in the morning and 75% in the afternoon. A 48-hour circadian sampling series in feeding fry was conducted at 911–934 dd (here named 920 dd) at the same time points and procedure as listed above.

### RNA extraction

Atlantic salmon heads were immersed in 10x volume of prechilled (-80°C) RNA later ICE (Invitogen, Carlsbad, CA, USA) for at least 48 h at -20°C before the brains were dissected out of the skulls. Total RNA was extracted from the salmon brains using TRI-reagent (Sigma, St. Louis, MO, USA) according to the manufacturer’s protocol. RNA quantity and quality were measured by Nanodrop ND-1000 spectrophotometer (Thermo Fisher Scientific, Waltham, MA, USA) before, and after, the total RNA was DNase I treated by using the TURBO DNA-free Kit (ThermoScientific, Waltham, MA, USA). The RNA integrity was checked using Agilent2100 Bioanalyzer with RNA 6000 Nano Kit (Agilent Technologies, CA, USA) revealing RNA integrity numbers (RIN) between 8.7–10.

### RNA sequencing

RNA samples (104 in total) were submitted to the Genomics Core Facility at the University of Bergen (Bergen, Norway) for RNA sequencing. Each sample was processed and sequenced using the Illumina Stranded mRNA Prep Ligation according to the manufacturers protocol on the Illumina NovaSeq6000 sequencing system (Illumina, Inc., San Diego, CA, USA) with 100 bp paired end reads per sample. All RNA sequencing data has been deposited to the European Nucleotide Archive accession number PRJEB74272.

### RNA sequencing analyzes

The RNA sequencing results were trimmed by Trimmomatic version 0.38 [36] and using STAR version 2.7.0 [37] the sequences were aligned to the published Atlantic salmon reference genome http://ftp.ensembl.org/pub/release-106/gtf/salmo_salar/. The output files from the aligner were processed by Samtools version 1.6 [38] and counts were generated using HTSeq version 0.11.2 [39]. Using DESeq2 version 1.26.0 [40] three normalized counts arrays were generated, namely (i) normalized counts for the 48 h circadian sampling series in salmon alevin (S1 Table), (ii) normalized counts for the 48 h circadian sampling series in salmon fry (S2 Table) and (iii) normalized counts for the 48 h circadian sampling series of alevin and fry together (S3 Table). The normalized count files were filtered by removing genes with a greater covariance than 100, and genes that had an average count of less than 5 within each sample. DESeq2 was also applied to do differential expression analyzes using Wald statistical model with a design that accounted for samples missing pituitary or/and pineal. Potentially missing pituitary and pineal were indicated by the number of normalized counts for candidate genes known to be pituitary of pineal specific. For pituitary proopiomelanocortin b (*pomcb*, ENSSSAG00000053129), thyrotropin βaα (*tshβaα*, ENSSSAG00000050784), growth hormone (*gh*, ENSSSAG00000055013), and somatolactin α (*slα*, ENSSSAG00000053861) was used based on studies in Atlantic salmon [41], grass carp [42] and Atlantic halibut [43]. For the pineal exorhodopsin (*exorh*, ENSSSAG00000006623) was used [44, 45]. Genes with counts less than 10 per comparison were not included in the DeSeq2 results and the adjusted p-value was set to < 0.05. Pathway enrichment analyzes was done in clusterProfiler version 1.13.0 [46] with the universal enrichment analyzer (enricher) with a pvalueCutoff = 0.05 and pAdjustMethod = “BH”. The resulting list of GO terms were analyzed in QuickGO [47, 48] revealing the association between GO terms, and the GeneIDs listed for each GO term were analyzed to reveal if associated GO terms shared GeneIDs. Venn diagrams were made in Venny v2.1 [49] and plots were created in the ggplot2 v3.3.6 package [50] in Rstudio [51].

### Identification of cycling gene expression profiles and annotations of genes

Daily rhythms were identified in the two 48 h circadian sampling series of alevin and fry by analyzing the normalized count arrays (S1 Table) and (S2 Table) by MetaCycle [52] v1.2.0 incorporating JTK_CYCLE [53]. The minimum and maximum length of the period were set using the default settings in JTK_CYCLE, 20 h and 28 h, respectively. The combinePvalue vas set to Bonferroni correction and the adjusted p-value threshold was analyzed for p < 0.1, p < 0.05, p < 0.01 and p < 0.001 with a minimum meta2d_Base of 10 counts (See S4 Table (720 dd) and S5 Table (920 dd)). The list of cycling genes with p < 0.05 and a minimum meta2d_Base of 10 counts was further analyzed by ClusterProfiler [46]. The 48 h circadian sampling series were also analyzed by CircaCompare [54] by plotting the circadian expression profile of clock genes that were shown to be cyclic by MetaCycle. The outputs of CircaCompare create a cosinusoidal curve with a parameterization that models and compare data from two circadian sampling series. In CircaCompare the alpha_threshold was set to 1, allowing to plot non-cyclic genes together with the significantly cyclic genes. The normalized count files in S1 and S2 Tables were used when the expression profile of alevins or fry was analyzed individually. The normalized count file in S3 Table was used when alevin and fry expression profiles were compared. The clock genes in Atlantic salmon have previously been identified [55] but in S6 Table a list of updated Ensembl GeneIDs is provided after the update of assembly and gene set from ICSASG_v2 to Ssal_v3.1. The annotation of genes in the pathway of circadian rhythm for Atlantic salmon (see S6 Table), except for the clock genes, was done by *in silico* analyzes, using BLASTN on NCBI [56] and Ensembl [57] with the information obtained from the KEGG pathway circadian rhythm in human (*Homo sapiens*), hsa04710 [58]. In addition, S6 Table includes proline and acidic amino acid-rich basic leucine zipper (PAR-bZip) factors (hepatic leukaemia factor (*hlf*) and thyrotroph embryonic factor (*tef*)) annotated in [59]. S6 Table also lists enzymes of the melatonin biosynthesis pathway from tryptophan to melatonin within the salmon KEGG pathway Tryptophan metabolism (sasa00380) [58]. Cycling genes in the pathway cell cycle were analyzed by using gene lists from KEGG pathway cell cycle (sasa04110) [58].

## Results

### Cycling of clock genes in endogenous and exogenous feeding Atlantic salmon

In the 48 h circadian sampling series the number of cycling genes analyzed by MetaCycle is given in S7 Table, showing that the number of genes with daily rhythms increases in the salmon brain during the first feeding period. At 720 dd, a total of 1090 genes were cycling with a period of 20–28 h (p < 0.05) while at 920 dd 1993 genes were cyclic. Genes cycling with a period of 20 h decreased from 720 dd to 920dd, while the number of cycling genes with a 24 h and 28 h period increased, with the 24 h constituting the majority. Pathway enrichment analyzes (p < 0.05) of the cyclic genes at 720 dd and 920 dd (based on periodicity (20 h, 24 h and 28 h) and all genes together, 1090 and 1993 genes, respectively) gave no overrepresented ontologies for 720 dd and only two ontologies were overrepresented for 920 dd, related to MAP kinase phosphatase activity (GO:0017017 and GO:0033549). Importantly, many of the circadian clock genes were shown to be cycling in the first feeding period (Table 1), while only three clock genes cycled (p < 0.05) in the brain of alevins dependent on endogenous feeding. These three genes belong to the *nr1d* family of clock genes and cycled with a period of 24 or 28 h. In the brain of fry, 20 clock genes cycled with a period of 24 h and six with a period of 28 h (p < 0.05). The cycling genes are members of all clock gene families, except the *csnk1e/d* family, where no genes cycled.

**Table 1:** Clock genes that cycled according to MetaCycle.

		720 dd				920 dd		
Gene name	20 h	24 h	28 h	Acrophase	20 h	24 h	28 h	Acrophase
*clock2a*	-	-	-	-	-	-	1,95E-02	-
*clock2b*	-	-	-	-	-	1,32E-07	-	00:00
*arntl1a*.*2*	-	-	-	-	-	8,72E-05	-	22:00
*arntl1b*.*1*	-	-	-	-	-	1,16E-03	-	22:00
*arntl1b*.*2*	-	-	-	-	-	4,58E-02	-	22:00
*arntl2c*	-	-	-	-	-	9,48E-05	-	06:00
*cry1a*.*1*	-	-	-	-	-	-	1,66E-03	-
*cry1a*.*2*	-	-	-	-	-	2,33E-02	-	22:00
*cry3b*.*1*	-	-	-	-	-	-	5,21E-05	-
*cry3b*.*2*	-	-	-	-	-	3,91E-09	-	22:00
*cry4*	-	-	-	-	-	2,53E-03	-	10:00
*per1a*.*1*	-	-	-	-	-	1,18E-07	-	06:00
*per1a*.*2*	-	-	-	-	-	1,39E-08	-	06:00
*per1b*	-	-	-	-	-	3,91E-06	-	08:00
*per2a*	-	-	-	-	-	9,48E-05	-	22:00
*per2b*	-	-	-	-	-	4,58E-02	-	00:00
*per3*	-	-	-	-	-	4,64E-03	-	14:00
*nr1d1a*	-	5,65E-03	-	20:00	-	-	2,53E-04	-
*nr1d2a*.*2*	-	-	-	-	-	-	6,42E-04	-
*nr1d4a*.*1*	-	-	-	-	-	-	2,53E-03	-
*nr1d4a*.*2*	-	-	-	-	-	1,05E-06	-	04:00
*nr1d4b*.*1*	-	2,93E-02	-	22:00	-	1,36E-02	-	00:00
*nr1d4b*.*2*	-	-	3,56E-03	-	-	6,20E-05	-	22:00
*rorca*.*2*	-	-	-	-	-	1,08E-03	-	22:00
*rorcb*.*1*	-	-	-	-	-	4,31E-06	-	22:00
*rorcb*.*2*	-	-	-	-	-	4,06E-04	-	22:00

Clock genes that cycle with a p-value < 0.05 and a minimum meta2d_Base of 10 counts at 720 dd and 920 dd. A period of 20 h, 24 h and 28 h was analyzed, and for genes with period 24 h the acrophase is given.

### The expression pattern of clock genes in endogenous and exogenous feeding Atlantic salmon

**Fig 1:**
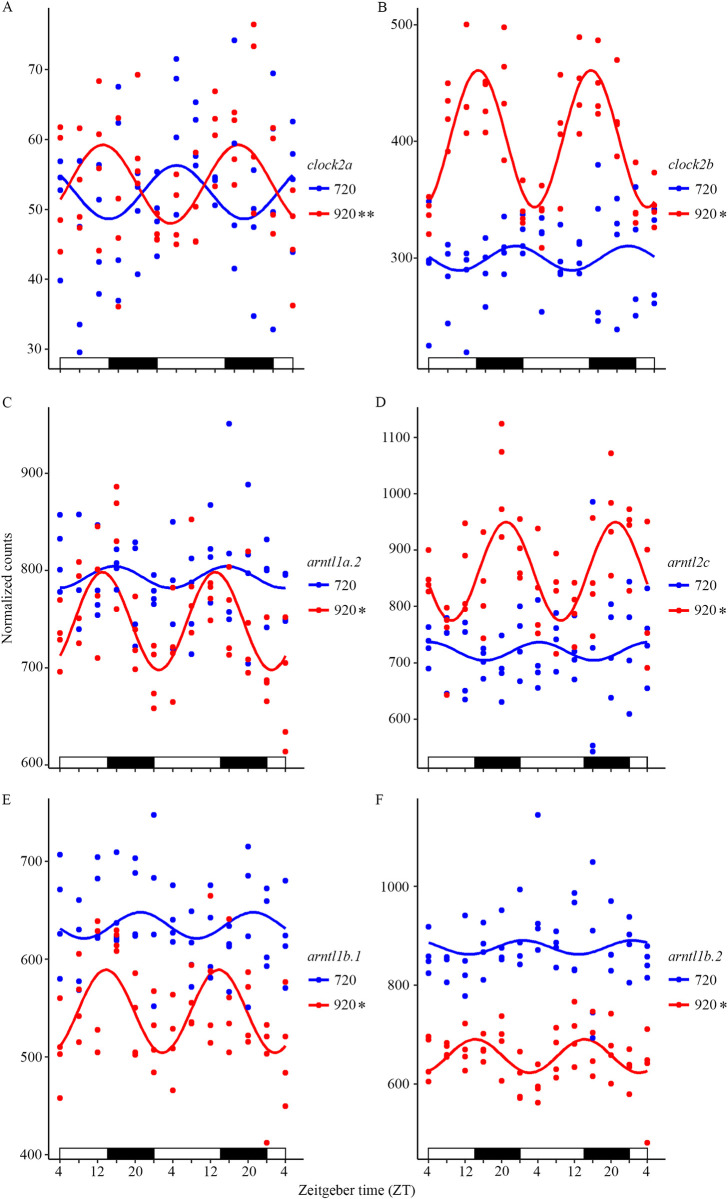
Brain expression profile of *clock* and *arntl* family members in endogenous (720 dd) and exogenous (920 dd) feeding Atlantic salmon. A) The *clock2a* expression at 920 dd was significantly cyclic. B) *clock2b* had a significantly high and cyclic expression at 920 dd compared to the non-cyclic at 720 dd. C) At 920 dd *arntl1a*.*2* had a significantly cyclic expression pattern. D) The significantly higher and cyclic expression of *arntl2c* at 920 dd, with an acrophase late in the night. E) *arntl1b*.*1* with a significantly lower and cyclic expression level at 920 dd. F) A significantly lower and cyclic expression level of *arntl1b*.2 at 920 dd compared to 720 dd. Plots are outputs of CircaCompare with a cosinusoidal curve drawn between the circadian sampling points. Bars at the x-axis indicate the light conditions. * 24 h, ** 28 h cycling period according to MetaCycle with p-value < 0.05.

The circadian expression profiles of the cyclic clock genes were analyzed by plotting in CircaCompare. Fig 1 shows the cyclic expression pattern at 920 dd of the core clock genes *clock* (Fig 1A and 1B) and *arntl* (Fig 1C–1F) family members together with the non-cyclic expression pattern of 720 dd alevins. The expression level of *clock2a* was similar between the two developmental stages (Fig 1A) but only the expression at 920 dd was significantly cyclic. For the significantly cycling *clock2b* (Fig 1B) and *arntl* genes (Fig 1C–1F), the expression level was significantly different between the two stages (DESeq2), with a clear amplitude in the expression at 920 dd. As shown in Table 1, the acrophases for all plotted genes at 920 dd, except for *arntl2c* at 06:00, were late in the light phase or early in the dark phase. Similar plots are given in S1 Fig (*cry*), S2 Fig (*per*), S3 Fig (*nr1d*) and S4 Fig (*rorc*), which compare the expression profile at 720 dd and 920 dd and shows the cyclic expression pattern at 920 dd. For the *nr1d* family members (S3 Fig) three genes were cyclic at both stages. For *nr1d1a* (S3A Fig) the expression at 720 dd was cyclic with a period of 24 h and an acrophase at 20:00, while at 920 dd the period was 28 h with a significantly higher expression level. For the cyclic *nr1d4b*.*1* (S3E Fig) and *nr1d4b*.*2* (S3F Fig) the expression profile was similar for the two developmental stages.

### Cycling of genes in the Kyoto Encyclopedia of Genes and Genomes (KEGG) pathway circadian rhythm

Table 2 shows cyclic genes in the KEGG pathway circadian rhythm [58], other than the clock genes, and cyclic proline and acidic amino acid-rich basic leucine zipper (PAR-zip) factors, the hepatic leukaemia factor (*hlf*) and thyrotroph embryonic factor (*tef*), at 720 dd and 920 dd (see S6 Table for annotations). The results show that six genes were cyclic at 720 dd but only two had a 24 h period, while at 920 dd, 14 genes were cyclic and ten of the genes had a period of 24 h. Among the genes that started to cycle with a daily rhythm of 24 h in fry, were genes that interact at sites containing D-boxes, the *cis*-regulatory elements for the PAR-zip transcription factors, including nuclear factor, interleukin‑3 regulated (*nfil3*) and several D‑box binding protein (*dbp*) genes. Also, several of the differentiated embryo chondrocyte (*dec*) genes were cyclic in the feeding fry. While several genes (protein kinase, AMP-activated (*prka*) and *cullin*) regulating the stability of the repressors Per and Cry (by phosphorylation and ubiquitylation E3 ligase complexes [8]), were also cyclic in alevins.

**Table 2:** Genes in the KEGG pathway circadian rhythm, hepatic leukaemia factors (*hlf*) and thyrotroph embryonic factors (*tef*) cycling according to MetaCycle.

		720dd				920dd		
Gene-name	20 h	24 h	28 h	Acro-phase	20 h	24 h	28 h	Acro-phase
*nfil3*	-	-	2.33E-02	-	-	1.91E-03	-	20:00
*dbpa*.*1*	-	-	-	-	-	7.83E-02	-	10:00
*dbpa*.*2*	-	-	-	-	-	2.36E-03	-	06:00
*dbpb*.*1*	-	-	-	-	-	9.38E-08	-	04:00
*dbpb*.*2*	-	-	-	-	-	3.25E-08	-	06:00
*dec1*.*1*	-	-	-	-	-	1.99E-04	-	18:00
*dec1*.*2*	-	-	-	-	-	-	1.66E-03	-
*dec2*.*1*	-	-	-	-	-	5.15E-11	-	06:00
*dec2*.*2*	-	-	-	-	-	5.64E-13	-	06:00
*prkaa1*.*1*	4.58E-02	-	-	-	-	-	-	-
*prkag1*.*1*	3.28E-02	-	-	-	4.10E-02	-	-	-
*cullin1*.*1*	-	-	-	-	-	1.45E-02	-	22:00
*cullin1*.*2*	-	2.61E-02	-	10:00	-	-	-	-
*cullin1*.*3*	-	-	1.16E-03	-	-	-	-	-
*hlf*.*3*	-	2.53E-03	-	00:00	-	7.46E-04	-	20:00
*hlf*.*4*	-	-	-	-	8.31E-03	-	-	-
*tef*.*3*	-	-	-	-	-	-	1.84E-02	-

Genes cycling with a p-value < 0.05 and a minimum meta2d_Base of 10 counts at 720 and 920 dd. A period of 20 h, 24 h and 28 h was analyzed, for genes with period 24 h the acrophase is given.

### Cycling of genes in the KEGG pathway cell cycle

Table 3 shows cyclic genes in the salmon KEGG pathway cell cycle [58] at 720 dd and 920 dd. At 720 dd, 14 genes cycled with a period of 20–28 h and, at 920 dd 11 genes cycled with a period of 20–28 h, with one gene *cdn1b* cycling in both developmental stages. Three cyclic genes had a period of 24 h at 720 dd (*ccne1*, *cullin1*.*2*, *chek2*), cycling with an acrophase in the light period. Five genes cycled at 920 dd (*cdk6*, *myca*, *cullin1*.*1*, *espl1*, *mad2a-like*), and all had an acrophase late in the light period or in darkness, except for *cdk6* with an acrophase at 12:00.

**Table 3:** Genes in the KEGG pathway cell cycle cyclic according to MetaCycle.

		720dd				920 dd		
Gene name	20 h	24 h	28 h	Acro-phase	20 h	24 h	28 h	Acro-phase
*tyrosine kinase*, *abl1*	3.56E-03	-	-	-	-	-	-	-
*glycogen synthase kinase-3 beta*, *gsk3b*	1.95E-02	-	-	-	-	-	-	-
*cyclin E1*, *ccne1*	-	1.28E-02	-	12:00	-	-	-	-
*cullin1*.*2*	-	2.77E-02	-	10:00	-	-	-	-
*cullin1*.*3*	-	-	1.25E-03	-	-	-	-	-
*chromatin licensing and DNA replication factor 1*, *cdt1*	2.77E-02	-	-	-	-	-	-	-
*14-3-3 protein zeta*	-	-	3.67E-02	-	-	-	-	-
*14-3-3 protein beta/alpha-A-like*	-	-	1.21E-02	-	-	-	-	-
*14-3-3 protein epsilon*	-	-	2.20E-02	-	-	-	-	-
*cell division cycle 23*, *cdc23*	4.33E-02	-	-	-	-	-	-	-
*MAU2 chromatid cohesion factor homolog*	-	-	3.56E-03	-	-	-	-	-
*Protein phosphatase 2*, *catalytic subunit*, *beta isozyme*, *pp2ab*	-	-	4.58E-02	-	-	-	-	-
*checkpoint kinase 2*, *chek2 *	-	1.45E-02	-	14:00	-	-	-	-
*tyrosyl-tRNA synthetase 2*, *cdn1b*	6.92E-04	-	-	-	-	-	4.58E-02	-
*cyclin dependent kinase 6*, *cdk6*	-	-	-	-	-	4.58E-02	-	12:00
*MYC proto-oncogene*, *bHLH transcription factor a*, *myca*	-	-	-	-	-	4.33E-02	-	00:00
*cyclin-dependent kinase inhibitor 2C*, *cdkn2c*	-	-	-	-	-	-	1.55E-03	-
*cullin1*.*1*	-	-	-	-	-	1.45E-02	-	22:00
*cell division cycle 26 homolog*, *cdc26*	-	-	-	-	1.45E-02	-	-	-
*extra spindle pole bodies like 1*, *espl1*	-	-	-	-	-	8.30E-03	-	18:00
*protein phosphatase 2 regulatory subunit B*,*alpha*, *ppp2r5a*	-	-	-	-	-	-	3.88E-02	-
*protein phosphatase 2*, *regulatory subunit B*, *gamma b*, *ppp2r5cb*	-	-	-	-	-	-	2.05E-03	-
*mitotic spindle assembly checkpoint protein mad2a-like*	-	-	-	-	-	4.83E-02	-	20:00

Genes cycling with a p-value < 0.05 and a minimum meta2d_Base of 10 counts at 720 and 920 dd. A period of 20 h, 24 h and 28 h was analyzed, for genes with period 24 h the acrophase is given.

### Circadian expression profile of important enzymes of the melatonin synthesis

**Fig 2:**
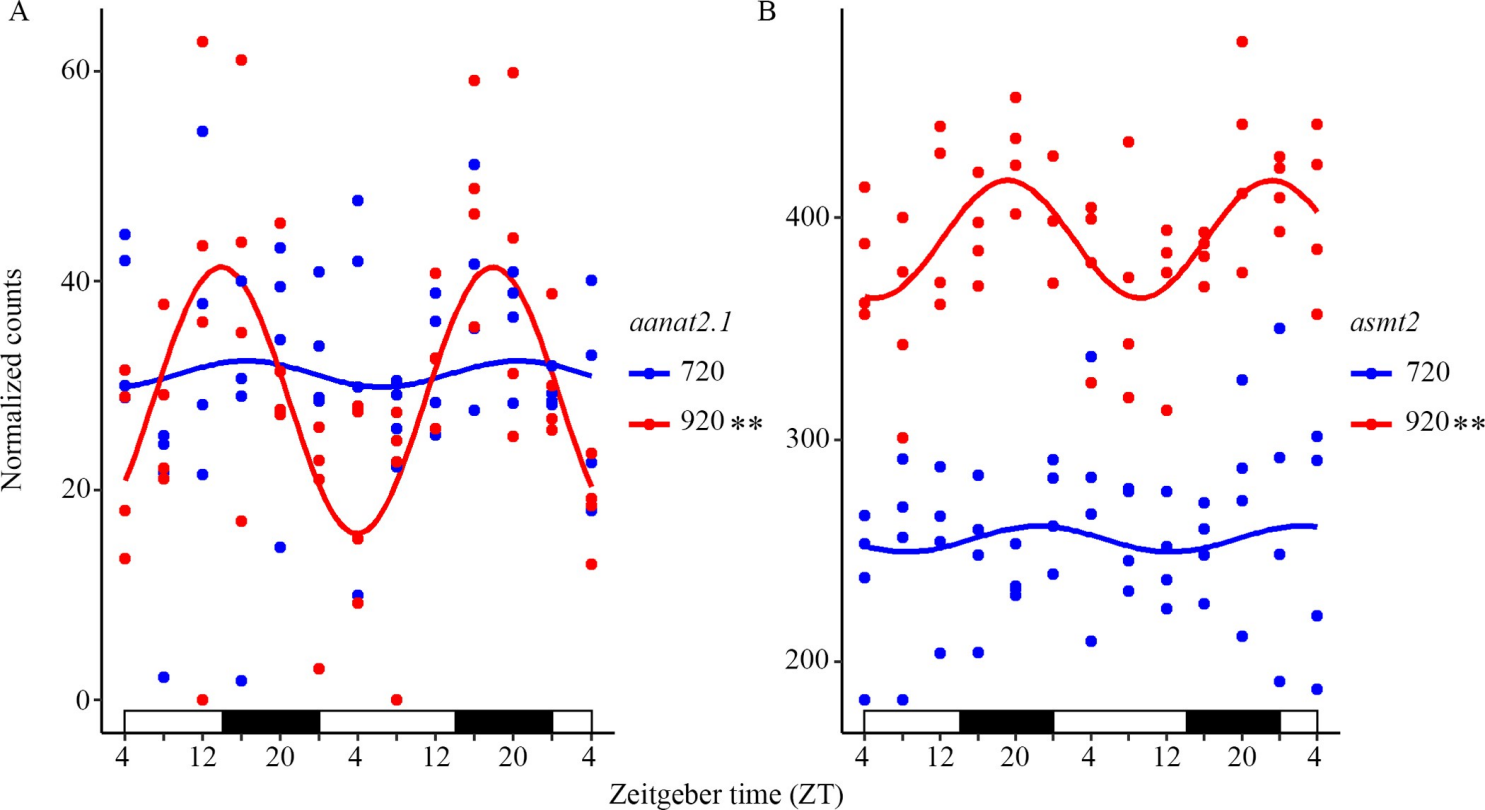
The expression profile of *aanat2*.*1* and *asmt2* in the brain of endogenous (720 dd) and exogenous (920 dd) feeding Atlantic salmon. A) The expression of *aanat2*.*1* at 920dd cycled significantly with a period of 28 h and had a similar expression level as in 720 dd. B) The expression of *asmt2* at 920 dd was significantly higher and cyclic, with a period of 28 h, at 920 dd compared to 720 dd. Plots are outputs of CircaCompare with a cosinusoidal curve drawn between the circadian sampling points. Bars at the x-axis indicate the light conditions. ** 28 h cycling period according to MetaCycle.

Important enzymes of the melatonin synthesis pathway were identified in the list from MetaCycle (See S6 Table for annotations), revealing that one of the Ss4R paralogues for the rate-limiting enzyme arylalkylamine N-acetyltransferase (*aanat*) and one of the melatonin-synthesizing enzyme acetylserotonin O-methyltransferase (*asmt*) had a cyclic expression pattern in fry. The *aanat2*.*1* and *asmt2* genes were both cycling with a period of 28 h, with acrophases at 22:00 and 04:00, respectively (S5 Table). The *asmt2* gene had a significantly higher expression level at 920 dd (DESeq2). Fig 2 shows the expression profile of the two genes plotted by CircaCompare for alevins and fry.

### Acrophase of the cyclic genes with a period of 24 h

**Fig 3:**
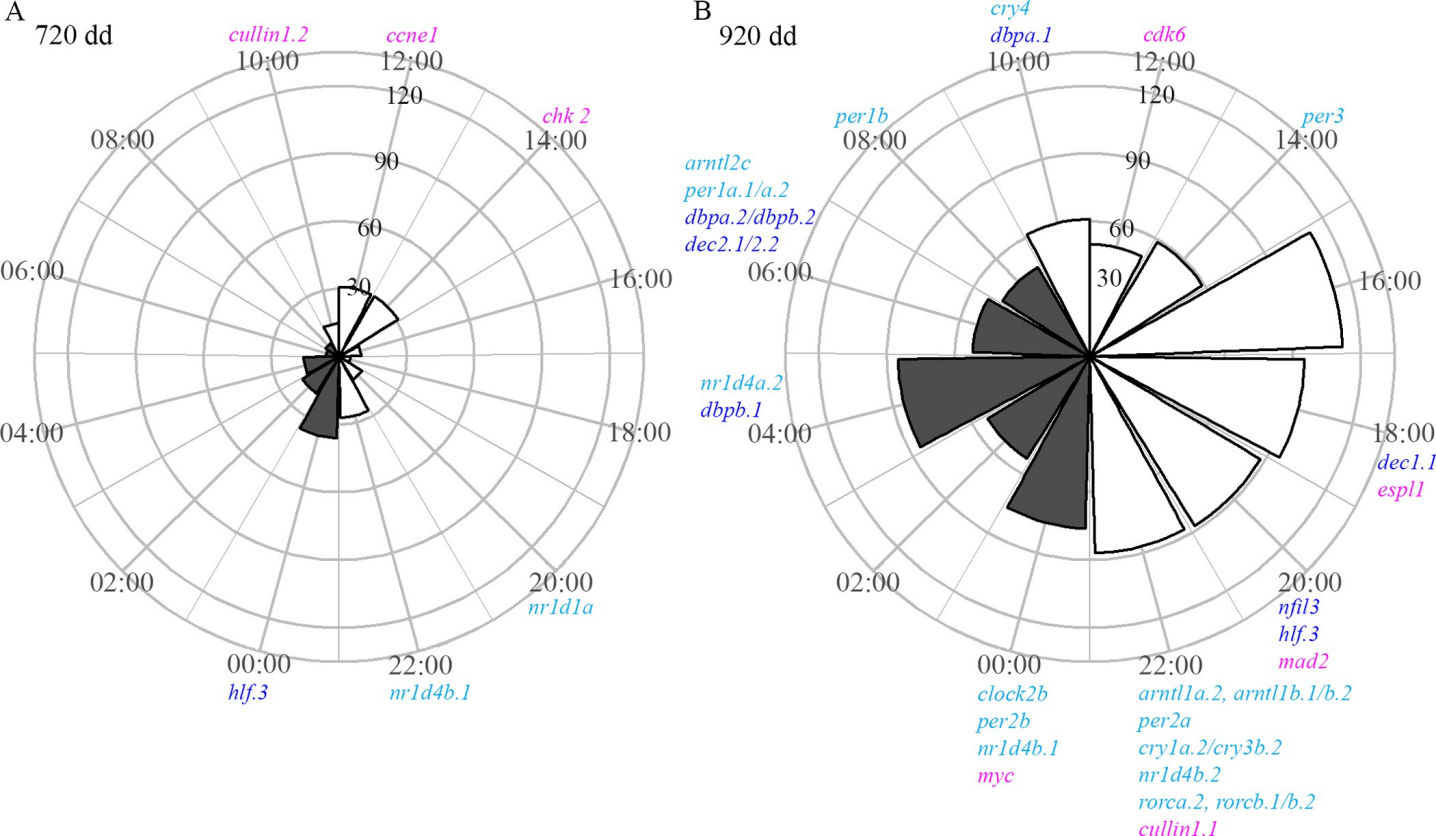
Acrophase of cyclic genes at endogenous and exogenous feeding according to MetaCycle. Rose plots of circadian cyclic genes at 720 and 920 dd with color coding of the bars indicating whether the acrophase occurs during the light period (white) or dark period (grey) (LD 14:10). The size of the bars indicates the number of genes with an acrophase at the given timepoint, with number shown with an increasing value of 30 for each circle. Clock genes (light blue), KEGG pathway circadian rhythm genes, hepatic leukaemia factor (*hlf*) (dark blue) and genes of the KEGG pathway cell cycle (magenta) are listed under the respective acrophase. A) Distribution by acrophase of 215 genes cyclic with a period of 24 h at 720 dd. B) Distribution by acrophase of 864 genes cyclic with a period of 24 h at 920 dd.

The distribution by acrophase of cyclic genes with a period of 24 h, according to MetaCycle, is shown in Fig 3, and illustrates an increase of cyclic genes with a period of 24 h in the feeding fry. Interestingly, at 22:00, in the transition between light and dark, and at 00:00, a total of 13 clock genes had a peak in the expression. Among the genes in the KEGG pathway cell cycle, three genes had an acrophase in the evening or early dark phase. At 06:00 and 08:00, late in the dark phase, several other clock genes and genes of the circadian rhythm pathway had a peak in their expression level. See S8 Table for an overview of the Ensembl gene IDs of genes with different acrophases.

### Differentially expressed genes in the circadian sampling series of alevin and fry brain

Differentially expressed genes (DEGs) between the same time point within a 48 h sampling series were analyzed to reveal if the data could be merged to a 24 h sampling series. The results gave few DEGs when comparing the same time point, except for 12:00 at 720 dd (S9 Table). The overall few DEGs allowed merging the samples from a time point (n = 12 for time point 12:00 and n = 8 for 16:00, 20:00, 00:00, 04:00 and 08:00) when performing the differential expression analyses. The resulting analyses, comparing the sampling points at 720 dd, gave only 37 DEGs, while a total of 2608 DEGs were found at 920 dd (Table 4 and S10 Table). Among the DEGs in the alevin brain, there were no clock genes, genes coding for enzymes of the melatonin synthesis, genes in the KEGG pathway circadian rhythm, *hlf* genes or *tef* genes, but one gene in the KEGG pathway of cell cycle. However, in the feeding fry several clock genes (see numbers in parenthesis in Table 4), genes in the KEGG pathway cell cycle and circadian rhythm and *hlf* genes were found among the DEGs (see annotations in S10 Table). In addition, analyzing genes in the melatonin synthesis pathway, *aanat2*.*1* was among the DEGs comparing 00:00 to 12:00 and 04:00 to 12:00 at 920 dd.

**Table 4:** Number of differentially expressed genes (DEGs) at 720 dd and 920 dd.

720 dd	12:00	16:00	20:00	00:00	04:00	08:00
12:00	-	9	2	0	0	0
16:00	-	-	4	9	0	2
20:00	-	-	-	5	1	0
00:00	-	-	-	-	4	1
04:00	-	-	-	-	-	0
08:00	-	-	-	-	-	-
920 dd	12:00	16:00	20:00	00:00	04:00	08:00
12:00	-	41(1)	67(3)	81(7)	160(5)	267(0)
16:00	-	-	0(0)	12(5)	212(7)	247(8)
20:00	-	-	-	37(3)	106(4)	283(12)
00:00	-	-	-	-	275(1)	468(9)
04:00	-	-	-	-	-	352(2)
08:00	-	-	-	-	-	-

The table shows the DEGs between time points within a developmental stage, revealing a difference in expression level of genes between time points in the feeding fry, with a total of 2608 DEGs obtained. Clock genes among the DEGs are indicated in parenthesis under 920 dd.

### Differential expression of the clock genes paralogues in the feeding fry

**Fig 4:**
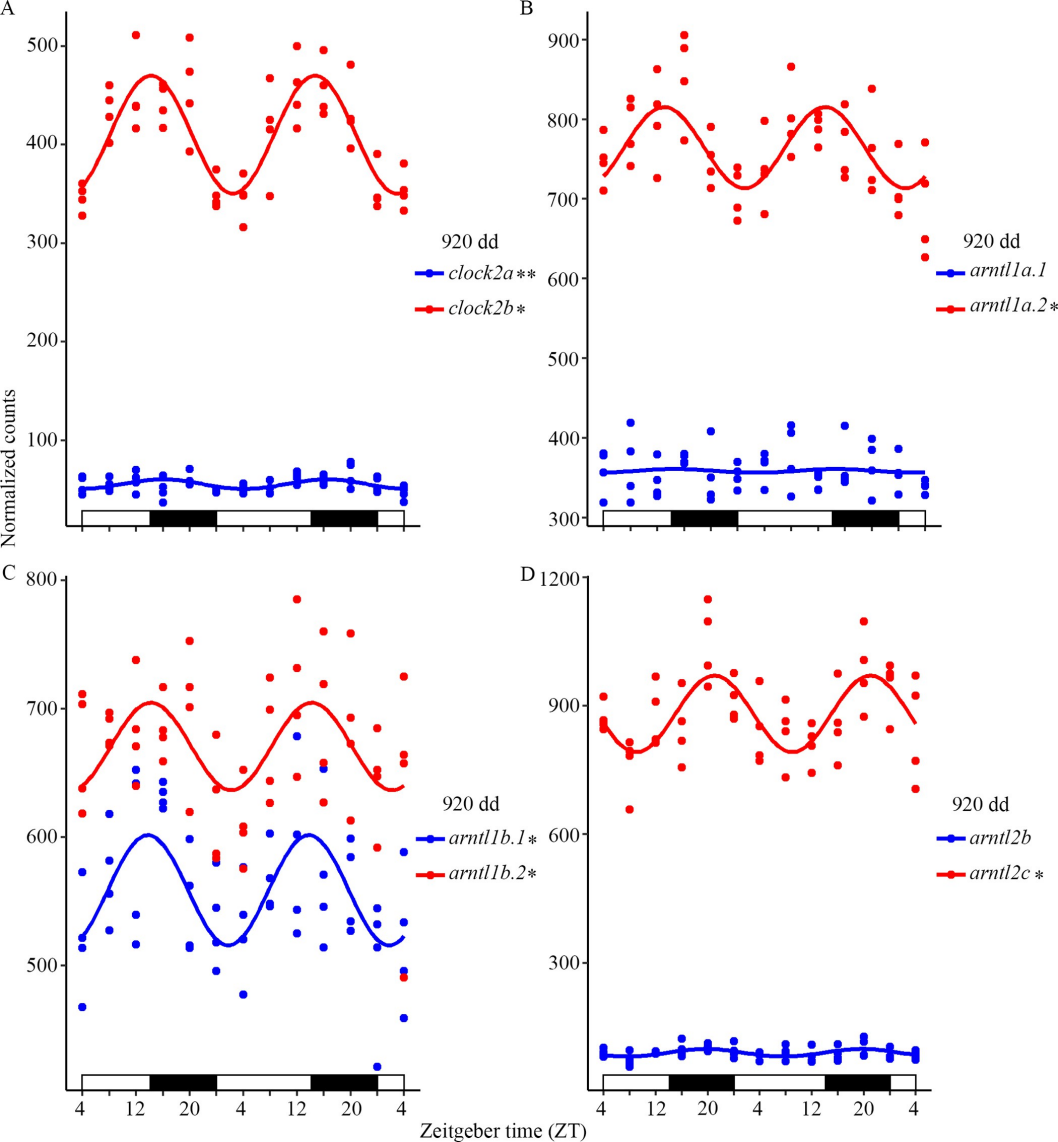
The circadian expression pattern of members of the *clock* and *arntl* family and their Ss4R paralogues at 920 dd. A) Both *clock2a* and *clock2b* had a significantly cyclic expression pattern but *clock2a* had a higher expression level. B) Only the paralogue *arntl1a*.*2* was significantly cyclic with a high expression level. C) Both Ss4R paralogues *arntl1b*.*1* and *arntl1b*.*2* were cycling with a period of 24 h and an acrophase 22.00. D) The paralogue *arntl2c* was significantly cyclic with an acrophase at 06:00, while the *arntl2b* expression was low. The acrophases for *clock2a*, *clock2b*, *arntl1a*.*2*, *arntl1b*.*1* and *arntl1b*.*2* were all late in the light phase or early dark phase. Plots are outputs of CircaCompare with a cosinusoidal curve drawn between the circadian sampling points. Bars at the x-axis indicate the light conditions. * 24 h, ** 28 h cycling period according to MetaCycle with p-value < 0.05.

The expression profile of cyclic clock genes and their respective Ss4R paralogues were compared by plotting them together in CircaCompare. Fig 4 shows the differential expression of members of the *clock* and *arntl* family at 920 dd. The Ss4R paralogues *clock2a* and *clock2b* were both significantly cycling, but with different periods and expression levels (Fig 4A). The expression level of *arntl1a*.*1* was non-cyclic and low compared to the significantly cycling Ss4R paralogue *arntl1a*.*2* (Fig 4B). The Ss4R paralogues *arntl1b*.*1* and *arntl1b*.*2* were similar, both significantly cyclic with a period of 24 h and an acrophase at 22:00 (Fig 4C). The *arntl2b* expression was non-cyclic and low compared to *arntl2c* which significantly cycled with a period of 24 h and had an acrophase at 06:00 (Fig 4D). Cycling of *cry* and *per* family members are shown in S5 Fig and *nr1d* and *rorc* family members in S6 Fig. Both *cry1a*.*1*/*cry1a*.*2* (S5A Fig) and *cry3b*.*1*/*cry3b*.*2* (S5B Fig) were significantly cycling but with different periods and acrophases. The Ss4R paralogues *per1a*.*1*/*per1a*.*2* (S5C Fig) both cycled with a period of 24 h and an acrophase at 06:00 while *per2a*/*per2b* (S5D Fig) had a period of 24 h but different acrophases. Only one Ss4R paralogue cycled significantly for *nr1d1a*/*nr1d1b* (S6A Fig), *nr1d2a*.*1*/*nr1d2a*.*2* (S6B Fig) and *rorca*.*1*/*rorca*.*2* (S6E Fig), while for *nr1d4a*.*1*/*nr1d4a*.*2* (S6C Fig), *nr1d4b*.*1*/*nr1d4b*.*2* (S6D Fig) and *rorcb*.*1*/*rorcb*.*2* (S6F Fig) both paralogues were significantly cycling. All *nr1d4* family members had an acrophase late in the light phase or in darkness. For *rorcb*.*1*/*rorcb*.*2* the expression level differed, but both genes had a period of 24h and an acrophase at 22:00.

### Difference between endogenous and exogenous feeding Atlantic salmon

**Fig 5:**
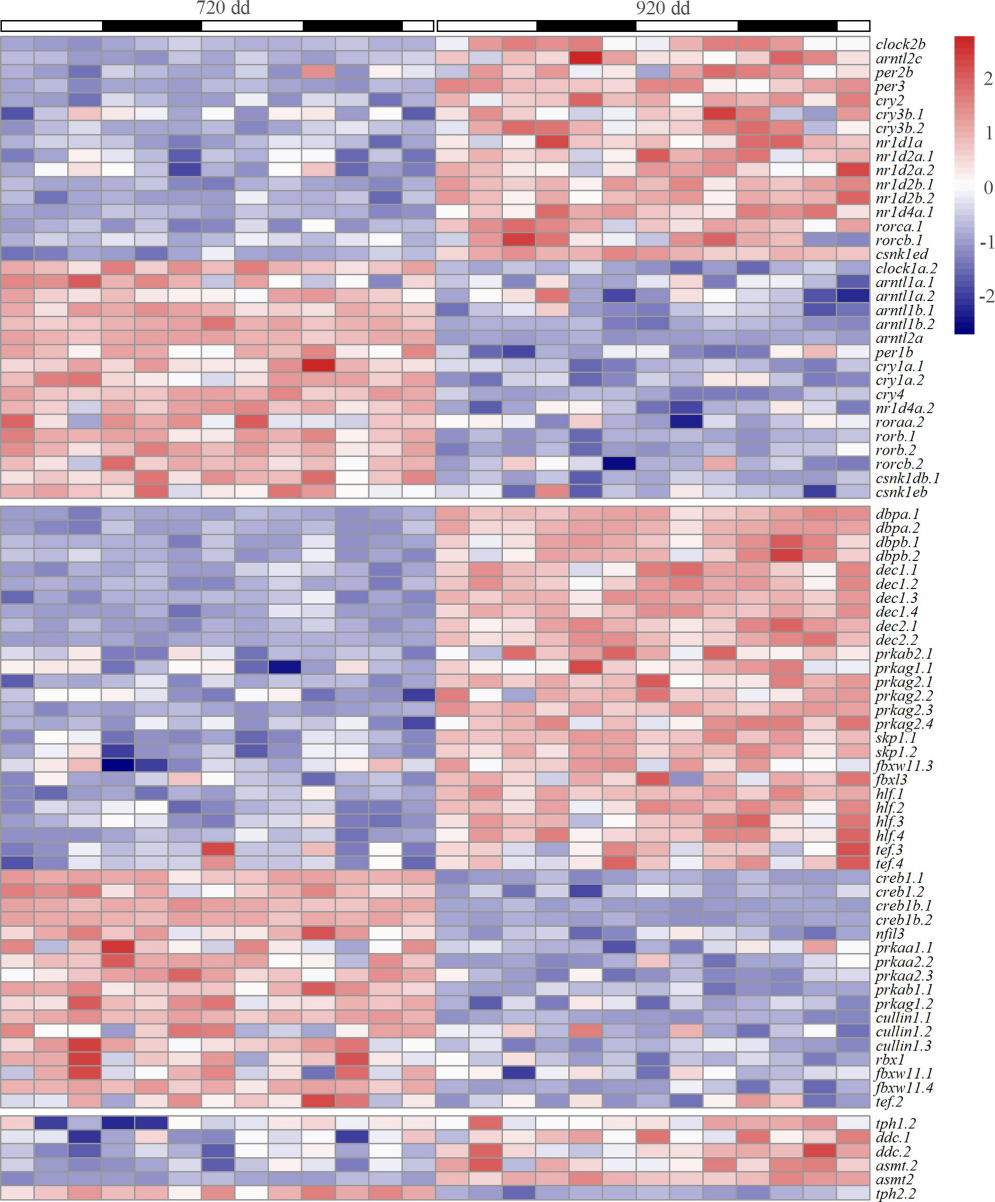
Heatmap of the differentially expressed genes. The heatmap shows DEGs among the clock genes, genes of the circadian rhythm KEGG pathway, *hlf*s and *tef*s and genes in the melatonin synthesis. Each sampling point is given by mean normalized counts and white or black bars indicate the light:dark period. The heatmap shows that in the brain of feeding fry dd, 16 clock genes were upregulated and 17 were downregulated, 26 genes of the circadian rhythm KEGG pathway, *hlf*s and *tef*s and genes were upregulated and 17 downregulated. Five genes of the melatonin synthesis were upregulated and one downregulated. The heatmap is scaled by row and genes are annotated as in S6 Table.

The DEGs between alevins and fry were analyzed comparing all samples at 720 dd and 920 dd. In total, 25984 genes were differentially expressed between the two stages (p < 0.05, baseMean > = 10), and among them 12974 genes were upregulated and 13010 downregulated in 920 dd compared to 720 dd (S7A Fig). Fig 5 shows a heatmap of clock genes, genes of the circadian rhythm KEGG pathway, *hlf*s and *tef*s, and genes of the melatonin synthesis that were differentially expressed, revealing that many of the genes in the S6 Table were either up- or downregulated between the two stages. Further, pathway enrichment analyses of the DEGs gave 222 upregulated and 125 downregulated GO terms in the class biological process, 104 upregulated and 55 downregulated GO terms in the class molecular function, and 92 terms upregulated and 51 downregulated GO terms in the class cellular compartment at 920 dd (S11 Table). Several of the upregulated GO terms at 920 dd were related to various metabolic processes (e.g., glycolytic process, lipid biosynthetic process, acetyl-CoA metabolic process and energy reserve metabolic process), homeostasis, the immune system, the actin cytoskeleton, organization of synapses and cell junctions, processes in the mitochondrion and Golgi apparatus, transport of various ions over membranes and enzyme activity (e.g., peptidase, kinase and lyase activity). Among the downregulated GO terms at 920 dd there were several related to the development of the embryo (e.g., brain development and Notch and Wnt signaling), the mitotic cell cycle and RNA processing and transcription. Analyses performed between sampling points at light and dark (720 dd light vs 920 dd light and 720 dark vs 920 dd dark) revealed fewer DEGs, compared to light and dark sampling points combined (S7A Fig). Nevertheless, the majority of DEGs were common (S7B, S7C Fig). The gene ontologies apparent in the comparisons between light samples at 720 and 920 dd and between dark samples at 720 and 920 dd are listed in S10 Table and the ontologies unique for the light samples or dark samples are shown in S7D, S7E Fig. Among the unique upregulated ontologies in 920 dd (light samples) several were related to metabolic processes, actin cytoskeleton, homeostasis, the immune system, and enzyme activity and among the unique downregulated terms in 920 dd (light samples) processes related to the cell cycle were abundant. In the dark samples, unique upregulated ontologies in 920 dd were e.g., neurogenesis and nervous system development, and downregulated terms were e.g., related to the Wnt signaling pathway and modification of histone.

## Discussion

The appearance of a functional timing system during early development of fish is not fully understood. It is still a question to what degree development and environment contribute to shape the appearance and functional activation of the clock system. In Atlantic salmon, living in the temperate zone with seasonal changes in light period, the circadian timing system also respond to these changes and regulate events like first feeding, smoltification and reproduction [33]. To determine a potential onset of circadian rhythmicity around the seasonal event first feeding, circadian sampling series of Atlantic salmon brains at the two developmental stages before and after exogenous feeding have been analyzed by RNA sequencing of 104 brains. The results provide a unique insight into how first feeding impacts the developing salmon brain, with a special emphasis on the clock genes and circadian rhythmicity. From an ecological point of view, it is very interesting that Atlantic salmon with its early stages in the river gravels, activates circadian rhythmicity for anticipating time then it needs to leave the gravel for feed.

### Establishment of a persistent circadian rhythmicity after first feeding

In medaka, the maturation of the circadian clock is dependent on the developmental stage and timing of hatching when the positive elements *clock* and *arntl* start to cycle [21]. Previous RNA sequencing of whole Atlantic salmon alevins has shown that the alevins are photoreceptive from an early developmental stage and that many clock genes are expressed early in development. However, only two genes are cyclic before exogenous feeding showing that the circadian clock is not oscillating at this developmental stage [31]. The study was done approx. 200 dd after hatching implying that a mature circadian clock is not linked to hatching as reported in medaka. The present study supports these observations, showing that only three clock genes were cyclic in the alevin brain. The three genes are members of the stabilizing accessory loop and one of the genes, *nr1d4b*.*2*, was cyclic in both studies. In contrast, in the feeding fry, 26 clock genes were significantly cycling (p < 0.05) demonstrating that circadian rhythmicity is established in Atlantic salmon when they change from a steady supply of nutrients from the yolk to a fry that needs to catch and ingest prey, indicating that this transition in development is important for the maturation of the circadian clock. The results showed that 20 of these genes cycled for a period of 24 h and the remaining six genes had a period of 28 h (by using the default period settings minimum period 20 h and maximum period 28 h in MetaCycle). In mice liver, genes have three major periods (8, 12 h, 24 h) [60] but, in this study, the genes with a period of 28 h are most likely a result of variance between individuals, rather than different periods of the clock genes. However, the use of default settings in MetaCycle allowed more cyclic genes to be included in the analysis, revealing rhythmicity in important key components of the clock and in clock-controlled genes such as important enzymes of the melatonin synthesis, that otherwise had been missed out. Thus, it is not likely that the periodicity of these genes is 28 h. A circadian sampling series of juvenile parr brains of Atlantic salmon, showed that 11 clock genes cycled with a 24 h period (p < 0.001) [55]. Comparing the results of exogenous feeding fry with those of the parr stage revealed several corresponding acrophases among the clock genes. For example, all cyclic genes in the *arntl1* family had an acrophase late in the light period or early dark period, while *arntl2c* had an acrophase late in the dark phase (06:00). Also, as seen in the parr stage, there was a phase shift in the peak (06.00) of *per1a* paralogs (*per1a*.*1*, *per1a*.*2*) compared to *per1b*, which peaked at 8.00 in fry and 08.30 in parr. A similar phase shift between the *per1a* and *per1b* genes has also been observed in zebrafish [61]. Surprisingly, the *per2* paralogues were demonstrated to have light-driven gene expression in zebrafish with acrophase early in the light phase [12, 13], opposed to Atlantic salmon brains, where the *per2* expression peaks during the dark phase in fry or in the transition from light to dark in parr. The comparable acrophases between the two stages of salmon indicate that a persistent circadian rhythm is established after first feeding.

### Potential zeitgebers acting as clock entrainment signals

In fish, daily cycles of light and temperature are two major zeitgebers entraining the biological clocks to the environment [62]. In this study, the light period was kept at LD14:10 throughout the experiment, providing the fish with a predictable zeitgeber of daily cycles. In addition, at transfer from egg incubation chambers to feeding tanks the light intensity was increased, compensating for an increased distance in the water column. However, both intensities are well above the above the light intensity of 2,6 x 10^−3^ W/m^2^ that is reported to be enough to reduce the melatonin level by 66% compared to darkness [63]. The temperature was kept constant over day and night, however, before first feeding the temperature was gradually raised to 11,5°C and then kept constant over the day and night. A temperature increase was done in accordance with standard rearing protocols, ensuring development of feeding behavior. Though, as the temperature was kept constant over day and night it was not considered as a zeitgeber entraining the biological clock in this study. Further, the fry was fed twice a day, and the restricted feeding might have been a synchronizer together with the LD cycle, as time-restricted feeding in mammals have been shown to affect the expression and rhythmicity of genes in several tissues [64]. Studies in fish have however shown that the feeding time has no effect on the rhythmicity of clock genes in the brain, only in the liver, indicating that the clock is entrain by feeding in the liver but not in the brain, where the rhythmic gene expression seems to be synchronized by LD cycles [65–67]. In addition, the acrophases of clock genes are comparable between salmon fry feed twice a day and salmon parr feed daily to satiation during the light period [55], indicating that the restricted feeding did not affect the entrainment of the clock in the brain of salmon.

### Acrophases of genes in the circadian clock mechanism

In zebrafish and sea bream, the core clock genes forming heterodimers are in phase with each other, *clock* and *arntl* are expressed at the beginning of the dark phase [68, 69] while the light-induced *per2* and *cry1a*, involved in light entrainment, have a peak early in the light phase [11, 12, 28]. In contrast, the nocturnally active mice have an increase of *Clock* and *Arntl1* early in the light phase and an increased expression of *Per* and *Cry* in the afternoon [8, 70]. Like zebrafish and sea bream, the salmon fry has cycling members of the *clock* and *arntl* families with an acrophase in dark or in the transition between light to dark (*clock2b* at 00:00 and *arntl1a*.*2*, *arntl1b*.*1* and *arntl1b*.*2* at 22:00). The genes *per2a*, *per2b*, *cry1a2* and *cry3b2* also had an acrophase in dark or in the transition between light to dark and were not in antiphase with *clock* and *arntl* as observed in zebrafish and sea bream. However, the results showed that *per1b* and *cry4* had an acrophase in the transition between dark to light and early in the light phase. As zebrafish Cry4 has been shown to not repress Clock-Arntl-mediated transcription and not to interact with Clock and Arntl [71], it is only speculation if these genes code for the negative elements in the circadian clock mechanism in salmon. In the stabilizing loop of the feeding fry, the cyclic members of transcription activators of *arntl1* all belonged to the *rorc* branch (*rorca*.*2*, *rorcb*.*1* and *rorcb*.*2*), with an acrophase at 22:00 in phase with members of the *arntl1* family. In comparison, the activators of *Arntl1* in mice have been shown to be differentially expressed and have different phases, e.g., *Rora* and *Rorb* were rhythmic with an expression peak during the light period in the suprachiasmatic nucleus while *Rorc* peaked at night in the liver, brown adipose tissue and kidney [72]. In mice, the Clock and Arntl1 heterodimer activates the expression of genes of the *Nr1d* family which again repress the expression of *Arntl1* leading to an antiphase expression of *Arntl1* and *Nr1d* [8, 73]. In salmon fry, *nr1d4a*.*2* (04.00) were in antiphase with the cyclic members of the *arntl1* family, while *nr1d4b*.*2* (22:00) were in phase. Taken together, several of the elements in the core and stabilizing loop were coordinated in exogenous feeding salmon, although the extra genome duplication in salmonids gives an additional complexity to the interpretation of the clock mechanism. Also, the extra whole genome duplication in salmonids has been suggested to allow paralogues of clock genes to diversify into non-circadian functions [59], here supported by differential expression and cycling/non-cycling of the clock paralogues in the fry brain.

### Cycling of genes in the KEGG pathway of circadian rhythm

Analyzes of the KEGG pathway circadian rhythm further supported an establishment of a persistent circadian rhythmicity in exogenous feeding fry, as several of the genes in the pathway were cyclic at this stage. Notably, the PAR-bZip factors (*dbp*, *hlf* and *tef*) together with the basic leucine zipper *nfil3*, genes coding for D-box binding proteins, had family members starting to cycle at 920 dd, with a 24 h period, except for *tef*.*3* with a period of 28 h. In mammals, the *Dpb*, *Hlf* and *Tef* were shown to bind and activate the promoter of *Per1* and to be expressed in advance/in phase with *Per1* in the suprachiasmatic nucleus [74, 75], while *Nfil3* was expressed in antiphase and the protein was shown to compete for the binding site with *Dpb*, *Hlf* and *Tef* [75]. As in mammals, the cyclic *dpb* genes in salmon fry were either expressed before or in phase with cyclic *per1* family members, except for *dbpa*.*1* which had an acrophase two hours after *per1b*. Further, *nfil3* was expressed in antiphase with *per1* genes but in phase with *hlf*.*3*. In mammals, Dec1 and Dec2 proteins are shown to repress Clock/Arntl1-induced activation of *Per1* promoter either by a direct protein-protein interaction with Arntl1 or/and by a competition for the E-box binding site [76]. In mice suprachiasmatic nucleus, expression peaked early in the day (*Dec1*) or middle of the day (*Dec2*) [76] and in a zebrafish peripheral cell line both *dec1* and *dec2* cycled with an acrophase two hours after the light was switched on [77]. In salmon, four *dec1* genes and two *dec2* genes were identified, and several of them started to cycle in feeding fry. Two of the *dec1* genes cycled, one cycled with a 24 h period and an acrophase at 18:00, while both *dec2* genes cycled with a period of 24 h and an acrophase at 06.00, corresponding to the acrophase of *per1a* genes. Compared to zebrafish, the expression pattern of *dec2* and *per1* genes was similar in salmon, peaking around the start of the light phase, in antiphase with *clock* and *arntl1* genes.

### Cycling of genes in the KEGG pathway of cell cycle

Circadian sampling series of whole feeding sea bream larvae have revealed clusters of genes related to the cell cycle peaking at different time points of the day, indicating a transcriptome profile resembling the cell cycle [28]. Three hours after the light was turned on, sea bream larvae had upregulation of key genes involved in the growth 1 phase/synthesis phase (G1/S) transition of the cell cycle such as *cyclin D*, cyclin dependent kinase 4 (*cdk4*), and proliferating cell nuclear antigen (*pcna*) important for DNA replication. Around 12 h later, when light was turned off, the sea bream larvae had upregulation of genes such as *cyclin A2* and *cdk1* involved in the transition from growth 2 phase (G2) to mitotic (M) phase and *cyclin B1* regulating the progression M phase of the cell cycle [28]. Among the significantly cyclic genes, with a period of 24 h in the alevin brain, there were three genes belonging to the KEGG cell cycle pathway of salmon [58]. The gene *cyclin E1* associating with cyclin dependent kinase 2 (*cdk2*) to promote G1/ S phase transition [78] had an acrophase at 12:00, while *cullin1*.*2* which is a part of the Skp1-Cul1-F-box-protein (SCF) ubiquitin ligase complex responsible for eliminating the Cyclin E/Cdk2 activity [79] had an acrophase at 10:00. In addition, checkpoint kinase 2 (*chek2*) which is a part of the DNA damage checkpoint and has a function in the induction of cell cycle arrest and apoptosis by DNA damage [80], had an acrophase at 14:00. Among the significantly cyclic genes at 920 dd, the expression of cyclin dependent kinase 6 (*cdk6*), forming a complex together with Cdk4 and Cyclin D allowing entry to the cell cycle [81], peaked at 12:00 early in the light phase, as seen for sea bream where *cyclin D* and *cdk4* peaked three hours after the lights were turned on. At 920 dd, *cullin1*.*1* a Ss4R paralogue of the cyclic *cullin1*.*2* at 720 dd, peaked at 22:00, indicating an active SKP1-CUL1-F-box-protein (SCF) ubiquitin ligase complex at the end of the light phase. The transcriptional regulator *c-myc* controlling expression of positive cell cycle regulators, important in the resting/gap phase (G0)/G1 transition [81, 82], was cyclic with an acrophase at 00:00 at 920 dd. Interestingly, the only overrepresented ontologies among the cyclic genes at 920 dd were related to MAP kinase phosphatase activity caused by genes of the family of dual specificity phosphatases which are a part of the KEGG MAPK signaling pathway influencing *c-myc* [81]. Also, *c-myc* among other cell cycle genes such as *cyclin D1*, *cyclin B1*, *cdk 1*, *wee1*, *p20* and *p21* are clock-controlled genes [83] and the heterodimer of Clock and Arntl1 has been shown to suppress *c-myc* transcription most likely through a E-box mediated reaction [84]. Among cyclic genes at 920 dd, two genes were related to the G2 and M phases of the cell cycle. The mitotic spindle assembly checkpoint protein *(mad2)*, a component of the mitotic checkpoint complex inhibiting anaphase initiation [81, 85] had an acrophase at 20:00 and the extra spindle pole bodies like 1 (*espl1*) gene, also known as “seperase” initiating the anaphase by an irreversible cleavage of the cohesin complex holding sister chromatids together [86], had an acrophase at 18:00. In compliance with feeding sea bream larvae, genes important in the G2 and M phase peaked late in the light phase. In addition, among the DEGs in the differential expression analyses between time points at 720 dd or 920 dd, only one gene in the KEGG cell cycle was apparent at 720 dd (*14-3-3 protein beta/alpha-A-like*) while eight genes were differentially expressed at 920 dd. Interestingly, one of these genes was the clock-controlled gene *wee1*, regulated by Clock and Arntl1 through the E-box [87] and an important inhibitor of progression into the M phase [81]. Other DEGs at 920 dd were mitotic checkpoint serine/threonine-protein kinase (*bub1*) and mitotic checkpoint protein (*bub3*) involved in the mitotic checkpoint complex. Taken together, these results indicate a clock-controlled cell cycle at exogenous feeding, with several genes important for cell cycle progression being cyclic or differentially expressed.

### Cycling of genes involved in the melatonin synthesis

In comparison to earlier studies of *aanat2* in salmonids [19, 20] the present RNA sequencing results allowed a detailed characterization of the salmon-specific paralogues, revealing a cyclic expression pattern of one of the two paralogues, *aanat2*.*1*. The results showed that *aanat2*.*1* started to cycle during the first feeding stage as seen for many of the clock genes and genes of the KEGG pathway of circadian rhythm. Even though *aanat2*.*1* cycled with a period of 28 h, the acrophase was indicated to be in the transition between light and dark at 22:00. These results correspond to *aanat* gene expression in most vertebrate’s pineal, with high levels during the night/dark phase and low levels during the day/light phase [16]. For example, the expression of *aanat2* in European sea bass (*Dicentrarchus labrax*) and catfish (*Clarias gariepinus*) peaked early in the night [20, 88]. Earlier *in silico* analyzes of the promoter region in Atlantic salmon have indicated a lack of E-box elements but revealed two complete E-boxes in the 5’UTR for *aanat2*.*1* and two imperfect E-boxes for *aanat2*.*2* [20]. The cyclic pattern of *aanat2*.*1*, but not of *aanat2*.*2*, may indicate that *aanat2*.*1* expression is regulated by Clock and Arntl1 through the complete upstream E-boxes. However, in dissimilarity to other teleosts [18], the lack of rhythmic melatonin release after a switch from LD cycles to constant darkness, still indicates that the circadian regulation of melatonin production is lost in salmonids. Further, the expression of *asmt2*, responsible for converting the N-acetylserotonin into melatonin, has earlier been indicated to be relatively constant throughout the day and night in fish [17], but was also shown to be cyclic in feeding fry of Atlantic salmon.

### The impact of first feeding on the developing brain

The developmental transition from endogenous to exogenous feeding is a change from an energetically closed system, where a steady supply of nutrients from the yolk is allocated for development, growth and metabolism, to a system where the nutrient supply depend on the success of foraging and ingestion of food [89]. During this transition, studies in rainbow trout have shown a turn from utilization of stored amino acids and lipids in the yolk for metabolism and growth, also with gluconeogenic activity, to a utilization of exogenous feeds where the metabolism includes glycolytic pathways and increased lipid storage through increased uptake and reduced utilization of fatty acids [90]. In line with this, the pathway enrichment analyses of DEGs comparing endogenous and exogenous feeding Atlantic salmon also showed upregulation of the glycolytic process in salmon fry. Upregulated DEGs in the glycolytic process term included important enzymes, such as hexokinase, phosphofructokinase and pyruvate kinase, which are involved in the breakdown of glycose to pyruvate. Further, the results showed that the protein catabolic process was among the downregulated terms in salmon fry, indicating a greater gluconeogenic activity before first feeding stage. Other upregulated GO terms at 920 dd included lipid biosynthetic process, acetyl-CoA metabolic process and energy reserve metabolic process, and as in rainbow trout, an increased lipid storage during exogenous feeding was observed. Further, in zebrafish, a switch to energy storage during the month-opening stage is observed [91]. In salmon fry, GO terms related to the immune system were upregulated and a similar enrichment of genes related to the immune system is shown in one-week old zebrafish [92]. Among the downregulated GO terms at 920 dd, there were several related to the development of the embryo, mitotic cell cycle and RNA processing and transcription. These results are in accordance with earlier developmental studies in Atlantic salmon, highlighting downregulation of ontologies related to the development in alevin that is functionally ready to commence exogenous feeding compared to earlier developmental stages [31].

In mammals, the metabolic rhythms are under control of the molecular clock mechanism and clock genes regulate cellular energy metabolism and body energy homeostasis through transcriptional control of metabolic genes. Among the clock genes and genes in the KEGG circadian rhythm pathway, *Arntl1*, *Dec1* and *Dec2* are suggested to be important in the interconnection between the clock mechanism and energy metabolism [93]. However, the function of the clock genes has also been shown to be altered due to changes in the energy balance [93] and, in mice, time-restricted feeding influence clock gene expression in a tissue-specific manner by changing the acrophase and amplitude [64], indicating a mutual regulation. Interestingly, the start of cycling of clock genes and genes in the KEGG circadian rhythm pathway, including *arntl* and *dec* genes, in the brain of salmon fry, indicates that the metabolic and circadian rhythms are intermingled. Thus, the life history transition of feeding is an important event for the onset of the circadian clock mechanism in Atlantic salmon.

## Conclusion

Taken together, the results revealed that circadian rhythmicity becomes established during the developmental transition first feeding in the Atlantic salmon brain, when the components of the molecular clock mechanism start to cycle. In addition, circadian clock output processes, such as the cell cycle and melatonin synthesis, are under circadian control in the feeding fry. Overall, this dataset provides a basis for understanding the impact of exogenous feeding on a developing teleost brain.

## Supporting information

S1 Fig The expression profile of *cry* family members at endogenous and exogenous feeding. A) Significantly cycling of *cry1a*.*1* at 920 dd. B) The *cry1a*.*2* expression was significantly cyclic at 920 dd. C) The significant cycling of *cry3b*.*1* at 920 dd compared to non-cyclic at 720 dd. D) Significantly cycling of *cry3b*.*2* at 920 dd. E) The significantly lower and cyclic expression of *cry4* in fry differed from the non-cyclic expression in alevins. Plots are outputs of CircaCompare with a cosinusoidal curve drawn between the circadian sampling points. Bars at the x-axis indicate the light conditions. * 24 h, ** 28 h cycling period according to MetaCycle with p-value < 0.05.
(TIF)

S2 Fig The expression profile of *per* family members at endogenous and exogenous feeding. A) *per1a*.*1* significantly cycled with a high amplitude at 920 dd. dd B) The amplitude was also high and significantly cyclic for *per1a*.*2* at 920 dd. C) The expression level of *per2a* was similar for the two developmental stages but only cyclic at 920 dd. D) The significantly cyclic *per2b* expression in fry. E) The significantly lower and cyclic expression of *per1b* at 920 dd compared to non-cyclic at 720 dd. F) The cyclic profile of *per3* at 920 dd. Plots are outputs of CircaCompare with a cosinusoidal curve drawn between the circadian sampling points. Bars at the x-axis indicate the light conditions. * 24 h cycling period according to MetaCycle with p-value < 0.05.
(TIF)

S3 Fig The expression profile of *nr1d* family members at endogenous and exogenous feeding. A) *nr1d1a* significantly cycled with different expression profiles at 720 dd and 920 dd. B) The expression of *nr1d2a*.*2* was significantly cycling at 920 dd. C) The expression level was significantly higher and cyclic at 920 dd for *nr1d4a*.*1*. D) Significantly cycling of *nr1d4a*.*2* in salmon fry. E) The expression profile of *nr1d4b*.*1* was similar and significantly cyclic for both developmental stages. F) The *nr1d4b*.*2* had a similar expression profile, but the gene significantly cycled with different periods in alevins and fry. Plots are outputs of CircaCompare with a cosinusoidal curve drawn between the circadian sampling points. Bars at the x-axis indicate the light conditions. * 24 h, ** 28 h cycling period according to MetaCycle with p-value < 0.05.
(TIF)

S4 Fig The expression profile of *rorc* family members at endogenous and exogenous feeding. A) *rorca*.*2* had a similar expression level at both developmental stages, significantly cyclin at 920 dd. B) *rorcb*.*1* significantly cycled with a high amplitude at 920 dd. C) The *rorcb*.*2* significantly cyclic at 920 dd. Plots are outputs of CircaCompare with a cosinusoidal curve drawn between the circadian sampling points. Bars at the x-axis indicate the light conditions. * 24 h cycling period according to MetaCycle with p-value < 0.05.
(TIF)

S5 Fig The circadian expression profile of members of the *cry* and *per* family and their Ss4R paralogues in feeding fry. A) The Ss4R paralogues *cry1a*.*1* and *cry1a*.*2* significantly cycled with different periods, acrophases and expression levels. B) *cry3b*.*1* and *cry3b*.*2* also cycled with different periods and acrophases. C) The *per1a*.*1* and *per1a*.*2* cycled with the same period and acrophase. D) Both paralogues cycle with the same period and with acrophases late in the light phase or dark phase. Plots are outputs of CircaCompare with a cosinusoidal curve drawn between the circadian sampling points. Bars at the x-axis indicate the light conditions. * 24 h, ** 28 h cycling period according to MetaCycle with p-value < 0.05.
(TIF)

S6 Fig The circadian expression pattern of members of the *nr1d* and *rorc* family and their Ss4R paralogues in feeding fry. A) The significantly cyclic *nr1d1a* shown together with the non-cyclic paralogue *nr1d1b*. B) *nr1d2a*.*1* was non-cyclic while *nr1d2a*.*2* was significantly cycling with a period of 28h and a high expression level. C) The Ss4R paralogues *nr1d4a*.*1* and *nr1d4a*.*2* were both significantly cycling with different periods. D) *nr1d4b*.*1* and *nr1d4b2* were both significantly cycling with a period of 24 h. E) The *rorca*.*1* was non-cyclic and lowly expressed compared to the significantly cyclic *rorca*.*2*. F) Both *rorcb*.*1* and *rorcb*.*2* were significantly cyclic but with a different expression level. Plots are outputs of CircaCompare with a cosinusoidal curve drawn between the circadian sampling points. Bars at the x-axis indicate the light conditions. * 24 h, ** 28 h cycling period according to MetaCycle with p-value < 0.05.
(TIF)

S7 Fig Differentially expressed genes between 720 dd and 920 dd and unique gene ontologies. A) Number of differentially expressed genes (DEGs) comparing all samples, only light (period) samples, dark (period) samples at 720 vs 920 dd, showing upregulated and downregulated in 920 dd (p < 0.05, baseMean > = 10). In all samples, 25984 DEGs, 12974 upregulated and 13010 downregulated. Light samples, 24578 DEGs, 12297 upregulated and 12280 downregulated and in dark samples 18366 DEGs, 8975 upregulated and 9390 were downregulated. B) Comparing the number of upregulated DEGs between all samples, light samples and dark samples. C) Comparing the number of downregulated DEGs between all samples, light samples and dark samples. D) Highlighting upregulated gene ontologies in light and dark samples. E) Highlighting downregulated gene ontologies in light and dark samples.
(TIF)

S1 Table Normalized counts for the 48 h circadian sampling series of Atlantic salmon alevins. (XLSX)

S2 Table Normalized counts for the 48 h circadian sampling series of Atlantic salmon fry. (XLSX)

S3 Table Normalized counts for the 48 h circadian sampling series of Atlantic salmon alevins and fry together. (XLSX)

S4 Table Cyclic genes in Atlantic salmon alevin brain. Result list from MetaCycle, sorted by p-value, including p-values < 0.1.
(XLSX)

S5 Table Cyclic genes Atlantic salmon fry brain. Result list from MetaCycle, sorted by p-value, including p-values < 0.1.
(XLSX)

S6 Table Annotations of clock genes, genes of the Kyoto Encyclopedia of Genes and Genomes (KEGG) pathway circadian rhythm, proline and acidic amino acid-rich basic leucine zipper (PAR-zip) factors and important elements of the melatonin synthesis. Ensembl GeneIDs after the update of assembly and gene set from ICSASG_v2 to Ssal_v3.1 for Atlantic salmon are provided.
(XLSX)

S7 Table The number of cycling genes in the Atlantic salmon brain given by MetaCycle. The number of cycling genes at 720 dd and 920 dd analyzed by different p-values (p < 0.1, 0.05, 0.01, 0.001) and periods (20 h, 24 h, 28 h).
(XLSX)

S8 Table Distribution of genes with different acrophases at 720 and 920 dd. The genes cycling with a period of 24 h according to MetaCycle are listed by Ensembl GeneIDs with their respective acrophase.
(XLSX)

S9 Table Number of differentially expressed genes (DEGs) within a time point. The number of DEGs was few within a time point both at 720 dd and 920 dd, except from time point 12:00 at 720 dd.
(XLSX)

S10 Table Differentially expressed genes (DEGs) between time points. The Ensembl GeneIDs for the comparisons are listed, including annotations.
(XLSX)

S11 Table Gene Ontology (GO) terms comparing 720 and 920 dd. GO terms sorted by up- and downregulation within the classes of biological process, molecular function, and cellular component. The comparisons are divided by the conditions All samples together (720 dd vs 920 dd), Light samples (720 dd vs 920 dd) and Dark samples (720 dd vs 920 dd).
(XLSX)
