# Supplementary figures and images for: Onset of circadian rhythmicity in the brain of Atlantic salmon is linked to exogenous feeding
Source: PLoS One. 2024 Nov 15;19(11):e0312911. doi: 10.1371/journal.pone.0312911 (PMC11567551; doi:10.1371/journal.pone.0312911)

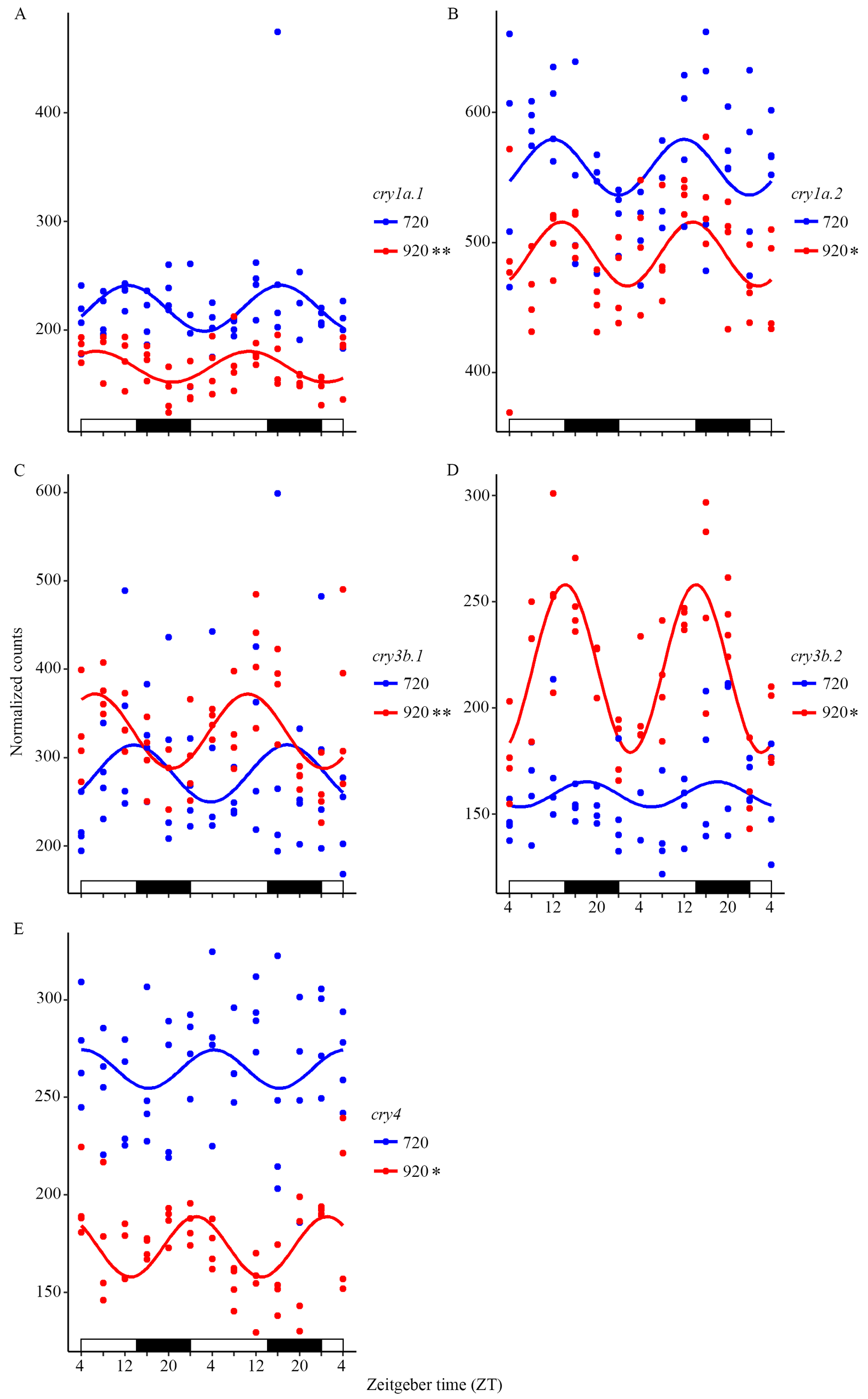

Supplement: S1 Fig — A) Significantly cycling of cry1a.1 at 920 dd. B) The cry1a.2 expression was significantly cyclic at 920 dd. C) The significant cycling of cry3b.1 at 920 dd compared to non-cyclic at 720 dd. D) Significantly cycling of cry3b.2 at 920 dd. E) The significantly lower and cyclic expression of cry4 in fry differed from the non-cyclic expression in alevins. Plots are outputs of CircaCompare with a cosinusoidal curve drawn between the circadian sampling points. Bars at the x-axis indicate the light conditions. * 24 h, ** 28 h cycling period according to MetaCycle with p-value < 0.05. (TIF) [file pone.0312911.s001.tif]

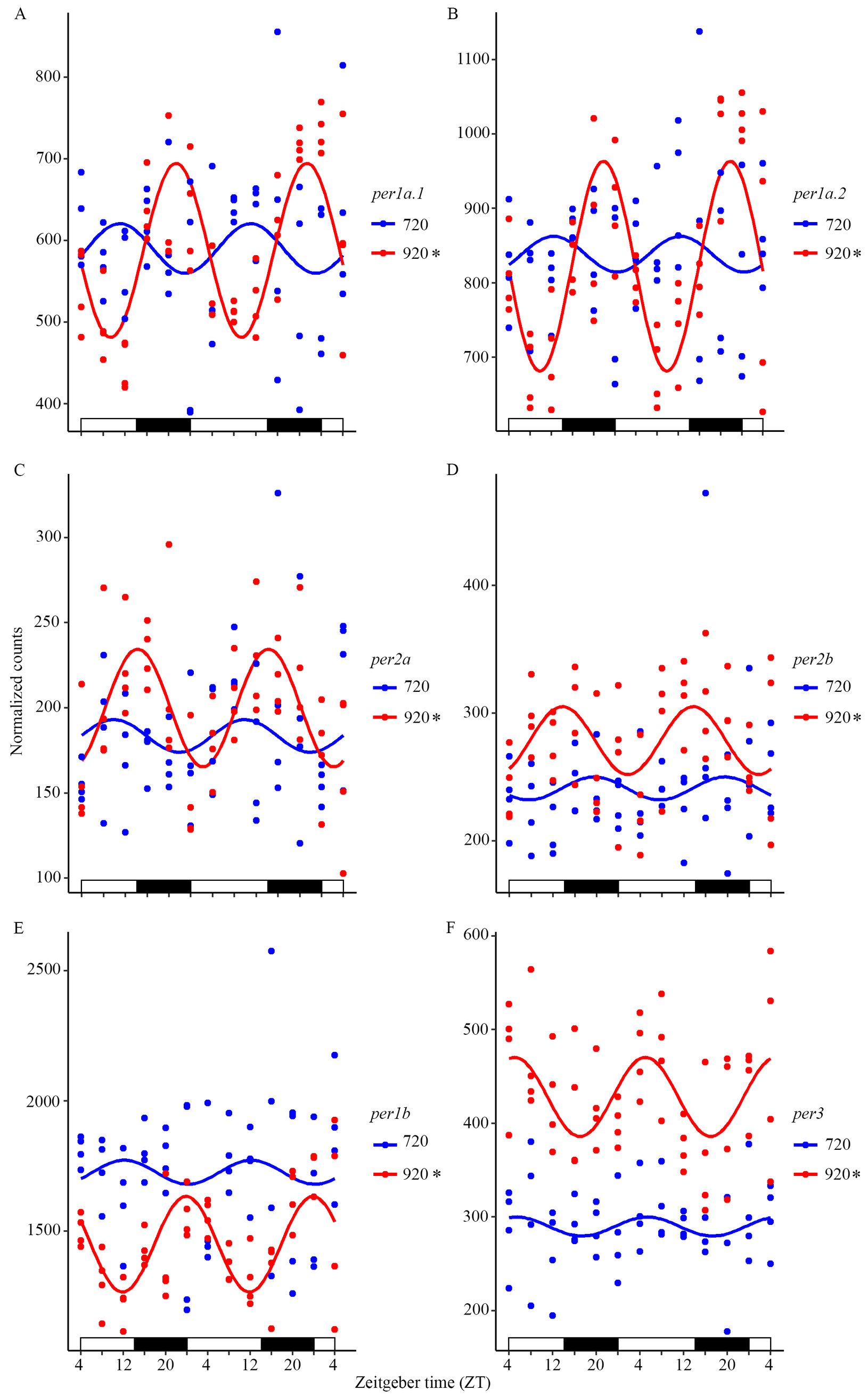

Supplement: S2 Fig — A) per1a.1 significantly cycled with a high amplitude at 920 dd. dd B) The amplitude was also high and significantly cyclic for per1a.2 at 920 dd. C) The expression level of per2a was similar for the two developmental stages but only cyclic at 920 dd. D) The significantly cyclic per2b expression in fry. E) The significantly lower and cyclic expression of per1b at 920 dd compared to non-cyclic at 720 dd. F) The cyclic profile of per3 at 920 dd. Plots are outputs of CircaCompare with a cosinusoidal curve drawn between the circadian sampling points. Bars at the x-axis indicate the light conditions. * 24 h cycling period according to MetaCycle with p-value < 0.05. (TIF) [file pone.0312911.s002.tif]

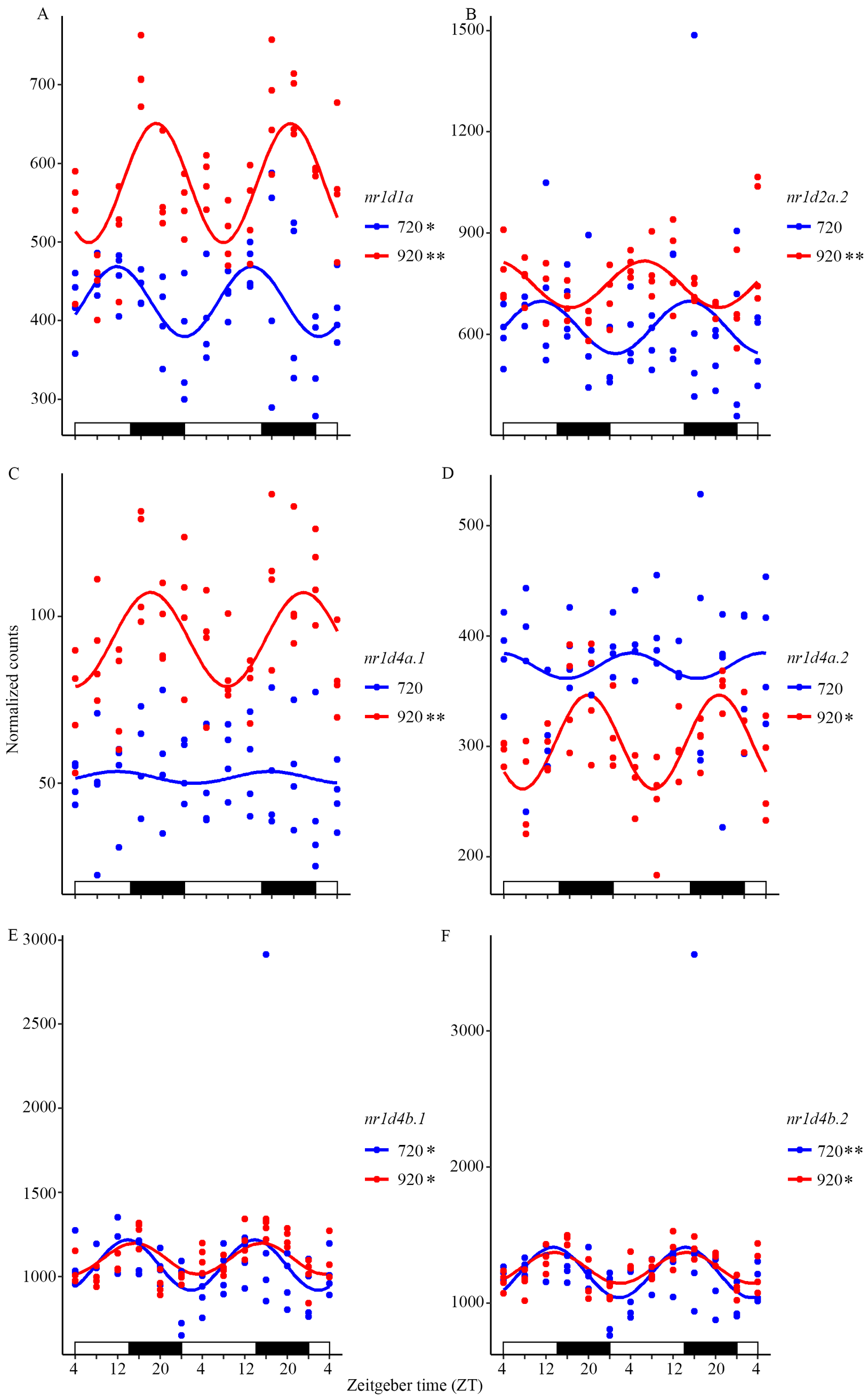

Supplement: S3 Fig — A) nr1d1a significantly cycled with different expression profiles at 720 dd and 920 dd. B) The expression of nr1d2a.2 was significantly cycling at 920 dd. C) The expression level was significantly higher and cyclic at 920 dd for nr1d4a.1. D) Significantly cycling of nr1d4a.2 in salmon fry. E) The expression profile of nr1d4b.1 was similar and significantly cyclic for both developmental stages. F) The nr1d4b.2 had a similar expression profile, but the gene significantly cycled with different periods in alevins and fry. Plots are outputs of CircaCompare with a cosinusoidal curve drawn between the circadian sampling points. Bars at the x-axis indicate the light conditions. * 24 h, ** 28 h cycling period according to MetaCycle with p-value < 0.05. (TIF) [file pone.0312911.s003.tif]

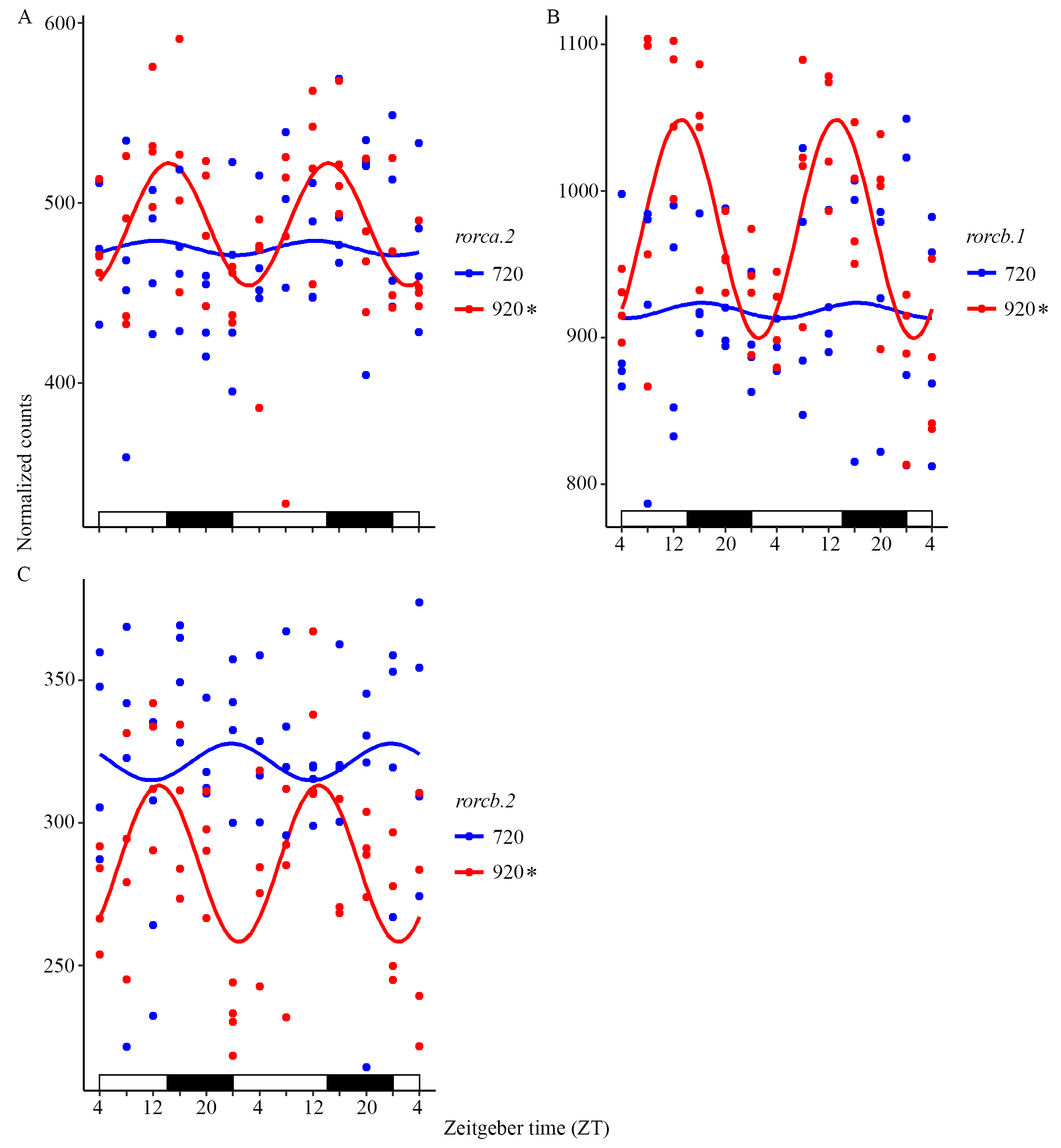

Supplement: S4 Fig — A) rorca.2 had a similar expression level at both developmental stages, significantly cyclin at 920 dd. B) rorcb.1 significantly cycled with a high amplitude at 920 dd. C) The rorcb.2 significantly cyclic at 920 dd. Plots are outputs of CircaCompare with a cosinusoidal curve drawn between the circadian sampling points. Bars at the x-axis indicate the light conditions. * 24 h cycling period according to MetaCycle with p-value < 0.05. (TIF) [file pone.0312911.s004.tif]

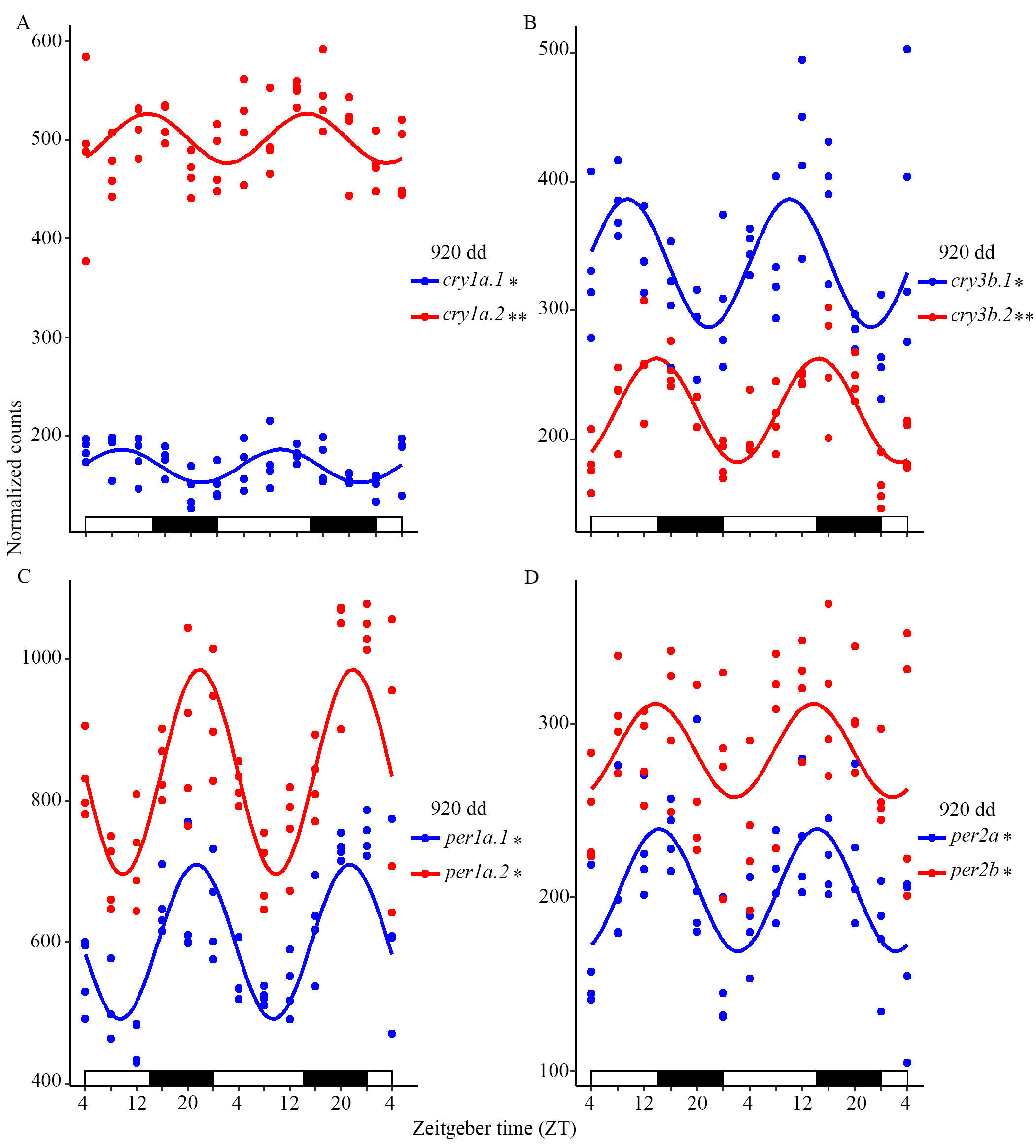

Supplement: S5 Fig — A) The Ss4R paralogues cry1a.1 and cry1a.2 significantly cycled with different periods, acrophases and expression levels. B) cry3b.1 and cry3b.2 also cycled with different periods and acrophases. C) The per1a.1 and per1a.2 cycled with the same period and acrophase. D) Both paralogues cycle with the same period and with acrophases late in the light phase or dark phase. Plots are outputs of CircaCompare with a cosinusoidal curve drawn between the circadian sampling points. Bars at the x-axis indicate the light conditions. * 24 h, ** 28 h cycling period according to MetaCycle with p-value < 0.05. (TIF) [file pone.0312911.s005.tif]

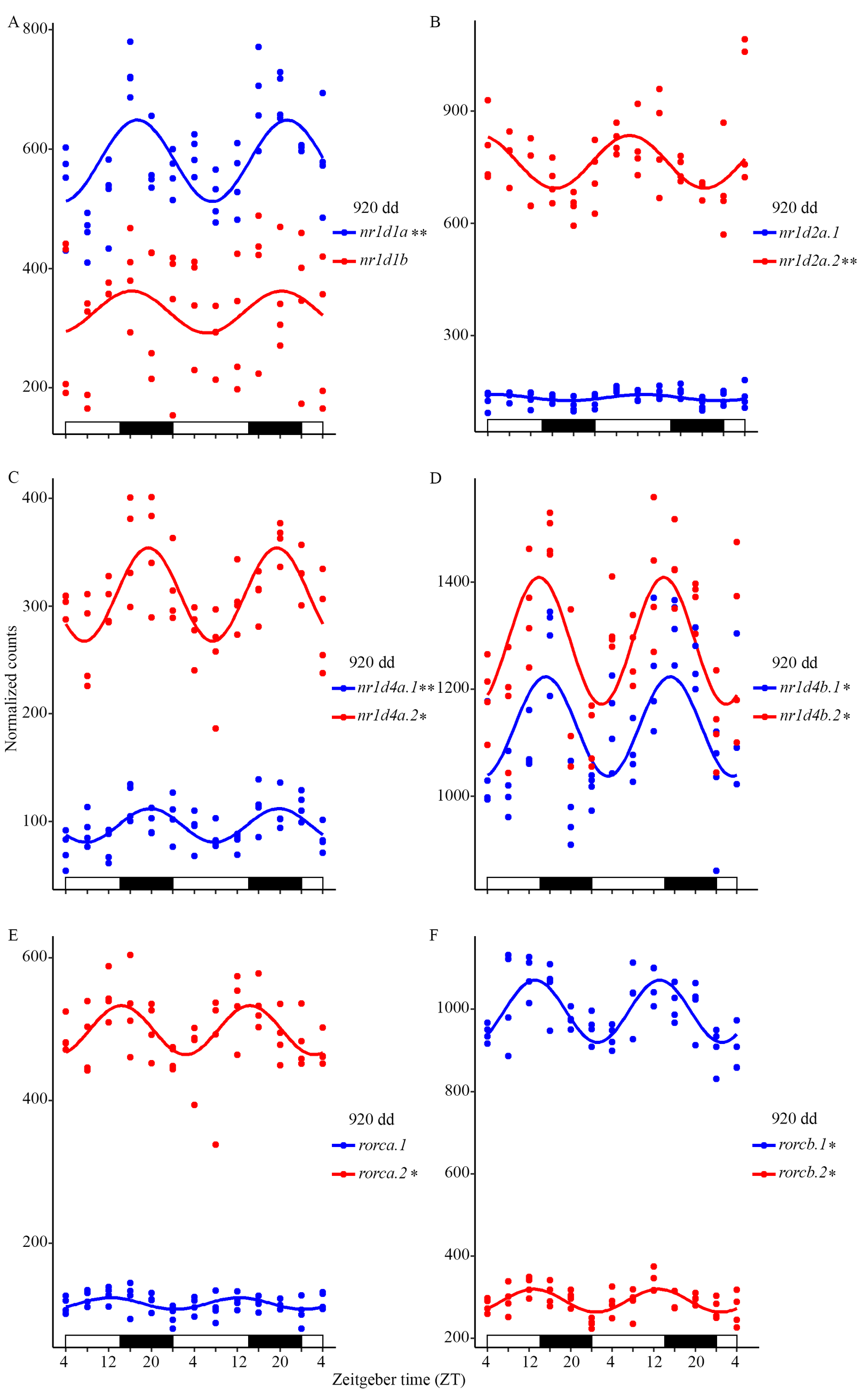

Supplement: S6 Fig — A) The significantly cyclic nr1d1a shown together with the non-cyclic paralogue nr1d1b. B) nr1d2a.1 was non-cyclic while nr1d2a.2 was significantly cycling with a period of 28h and a high expression level. C) The Ss4R paralogues nr1d4a.1 and nr1d4a.2 were both significantly cycling with different periods. D) nr1d4b.1 and nr1d4b2 were both significantly cycling with a period of 24 h. E) The rorca.1 was non-cyclic and lowly expressed compared to the significantly cyclic rorca.2. F) Both rorcb.1 and rorcb.2 were significantly cyclic but with a different expression level. Plots are outputs of CircaCompare with a cosinusoidal curve drawn between the circadian sampling points. Bars at the x-axis indicate the light conditions. * 24 h, ** 28 h cycling period according to MetaCycle with p-value < 0.05. (TIF) [file pone.0312911.s006.tif]

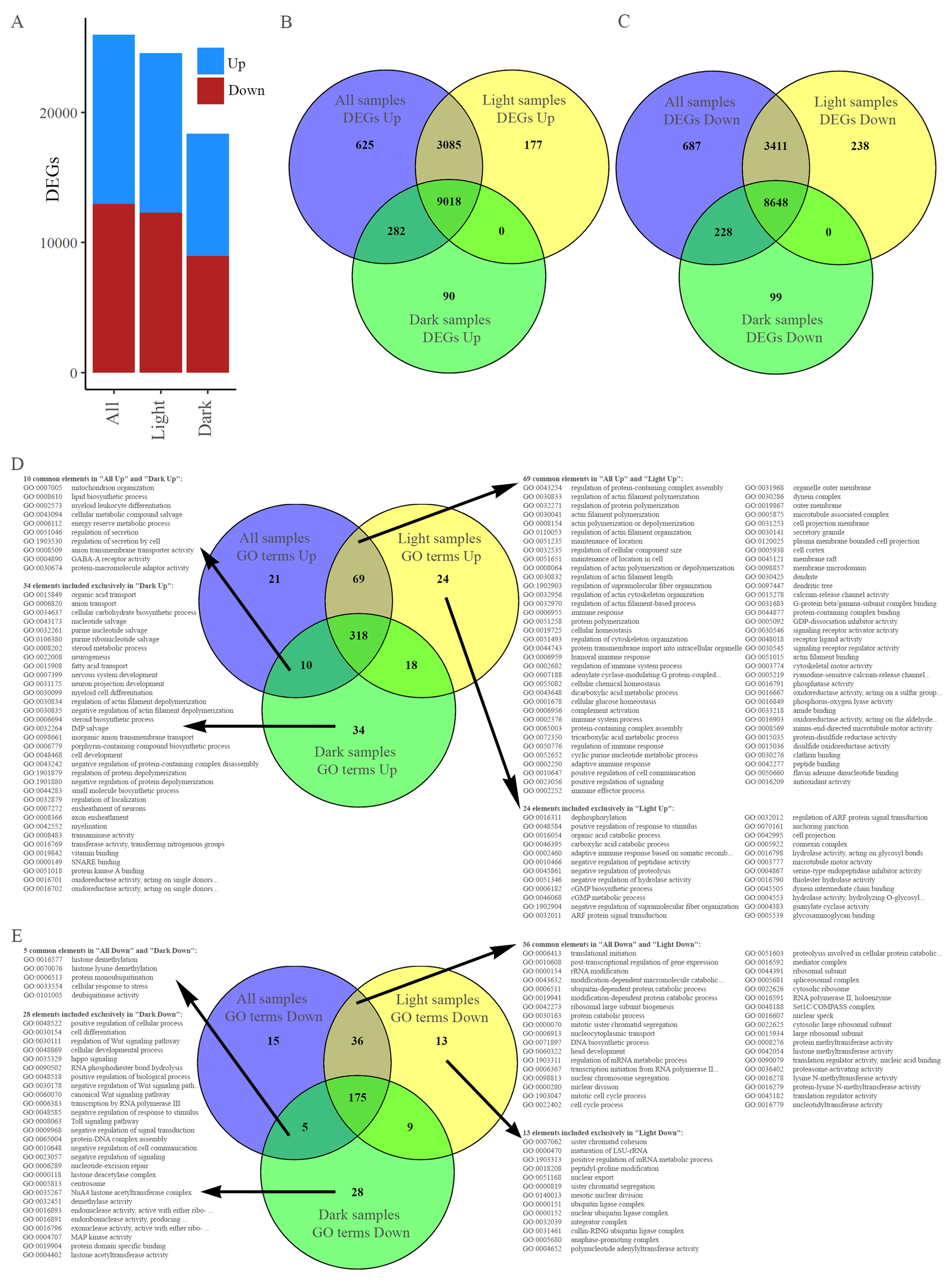

Supplement: S7 Fig — A) Number of differentially expressed genes (DEGs) comparing all samples, only light (period) samples, dark (period) samples at 720 vs 920 dd, showing upregulated and downregulated in 920 dd (p < 0.05, baseMean > = 10). In all samples, 25984 DEGs, 12974 upregulated and 13010 downregulated. Light samples, 24578 DEGs, 12297 upregulated and 12280 downregulated and in dark samples 18366 DEGs, 8975 upregulated and 9390 were downregulated. B) Comparing the number of upregulated DEGs between all samples, light samples and dark samples. C) Comparing the number of downregulated DEGs between all samples, light samples and dark samples. D) Highlighting upregulated gene ontologies in light and dark samples. E) Highlighting downregulated gene ontologies in light and dark samples. (TIF) [file pone.0312911.s007.tif]
